# Automation and sectoral reallocation

**DOI:** 10.1007/s13209-021-00240-w

**Published:** 2021-07-30

**Authors:** Dennis C. Hutschenreiter, Tommaso Santini, Eugenia Vella

**Affiliations:** 1 Universitat Autònoma de Barcelona and Barcelona GSE, Barcelona, Spain; 2 Athens University of Economics and Business and Fundació MOVE, Athens, Greece

**Keywords:** Automation, Manufacturing, Services, Sectoral reallocation, Participation, Matching frictions, Vacancy creation, Productivity, E24, O14, O33, J22, J23

## Abstract

Empirical evidence in Dauth et al. (J Eur Econ Assoc, 2021) suggests that industrial robot adoption in Germany has led to a sectoral reallocation of employment from manufacturing to services, leaving total employment unaffected. We rationalize this evidence through the lens of a general equilibrium model with two sectors, matching frictions and endogenous participation. Automation induces firms to create fewer vacancies and job seekers to search less in the automatable sector (manufacturing). The service sector expands due to the sectoral complementarity in the production of the final good and a positive wealth effect for the household. Analysis across steady states shows that the reduction in manufacturing employment can be offset by the increase in service employment. The model can also replicate the magnitude of the decline in the ratio of manufacturing employment to service employment in Germany between 1994 and 2014.

**Supplementary Information:**

The online version supplementary material available at 10.1007/s13209-021-00240-w.

## Introduction

As a result of improved capabilities and falling production costs, the global operational stock of industrial robots rose by about 65% within five years (2013–2018). The Covid-19 pandemic crisis is expected to accelerate further the speed of automation (see, e.g., Dolado et al. (2020) and Leduc and Liu (2020a)). In addition to the potentially significant implications for labor markets, recent evidence reveals that higher exposure to robot adoption has increased support for nationalist and radical right parties in Western Europe (Anelli et al. (2020)).

Academic and policy debates have focused on whether robots cause job displacement or job creation in the economy. On the one hand, a negative displacement effect arises from the fact that robots can outperform workers in some tasks. For instance, Acemoglu and Restrepo (2020) find that each robot installed in the USA replaces six workers. On the other hand, a positive productivity effect occurs because machines can help fewer workers produce more output, which increases labor demand. In this vein, the seminal work by Graetz and Michaels (2018) finds, using industry-level data from 17 countries, that cumulative changes in robot adoption from 1993 to 2007 boost labor productivity and raise wages.^1^

Notably, the adjustment in other parts of the economy—for instance, when other sectors expand to absorb the labor freed from robot adoption—has received little attention so far. According to empirical evidence for Germany in Dauth et al. (2021), industrial robots have changed the composition but not the aggregate size of employment, with job gains in services offsetting the negative impact on manufacturing employment. shows the evolution of the employment shares and labor compensation (as a share of value added (VA)) in the two sectors along with the stock of industrial robots. Germany is the country with the highest robot density in Europe (see Fig. 2).^2^

**Fig. 1:**
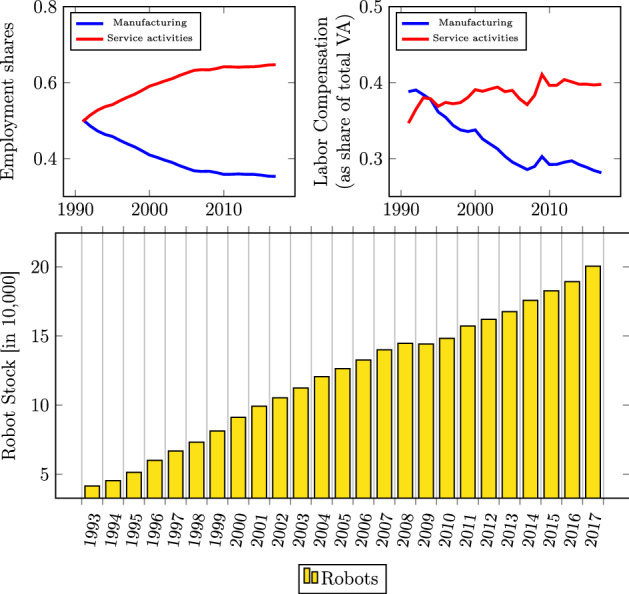
Industrial robots, employment and employees’ compensation in Germany. Note: Employment shares and labor compensation are calculated from EUKLEMS data. Data on the stock of industrial robots are from the International Federation of Robotics (IFR)

**Fig. 2:**
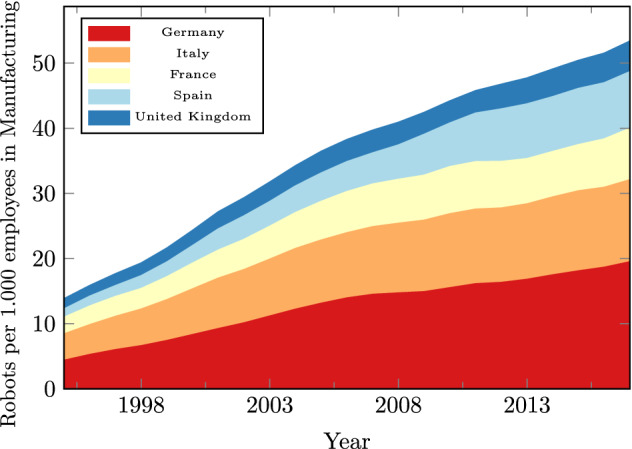
Industrial robot density in the manufacturing sector of European economies. *Note* Data on the stock of industrial robots are from the International Federation of Robotics (IFR). We define the manufacturing sector as the aggregate of Industries A-F in the German WZ08 (NACE Rev. 2) industry classification

To rationalize the empirical evidence on the automation-driven sectoral reallocation of labor in Germany, we develop a general equilibrium model with two production sectors, a labor market participation choice and matching frictions.^3^ Automation increases the capital intensity of the technology in the manufacturing sector as motivated by the microfoundations derived by Acemoglu and Restrepo (2018), consistently with empirical observations, and close in spirit to Bergholt et al. (2021).^4^ The presence of the extensive margin in our model is motivated by recent literature highlighting the negative effect of automation on participation, both in the short run and the long run (see, e.g., Grigoli et al. (2020), Lerch (2020), and Jaimovich et al. (2020)). Overall, the adjustment of sectoral labor markets in response to automation takes place in the model through three channels: (i) job creation, (ii) sector-specific search of unemployed job seekers, and (iii) participation. Since our representative household model is capable of rationalizing the empirical evidence mentioned above, we abstract from heterogeneous households for simplicity.

Calibrating the model for Germany and focusing on long-run analysis, we show that automation induces firms to create fewer vacancies and job seekers to search less in the robot-exposed sector (manufacturing). The model is able to replicate the empirical evolution of the sectoral employment shares and labor compensation in manufacturing and services (Fig. 1). Labor demand in services increases due to two effects. Firstly, an increase in automation decreases the marginal cost in manufacturing in the long run. The two sectoral goods are gross complements in the production of the final consumption good. Therefore, the positive income effect on services dominates the negative substitution effect due to a decrease in the relative price of manufacturing caused by automation. This result is consistent with the model of Acemoglu and Restrepo (2020), where higher robot adoption increases demand for complementary inputs. Additionally, as more capital is accumulated in the steady state due to the exogenous increase in automation, the demand for the aggregate good increases (positive wealth effect). We show through analysis across steady states that the reduction in manufacturing employment can be offset by the increase in service employment, thus leaving aggregate employment unaffected, in line with the empirical findings of Dauth et al. (2021).

In the model, structural change due to automation leads to a reallocation of workers from the manufacturing sector to the service sector. Furthermore, the model generates a negative effect of automation on participation in line with the literature. As we seek to explain how total employment can consequently remain constant, the presence of unemployment is crucial to generate the patterns observed in the data. Without unemployment and endogenous participation, that would be true by construction.

Our analysis highlights vacancy creation (labor demand) as the primary channel through which the two labor markets adjust to automation. The elasticities of substitution between capital and labor in manufacturing production and between automatable (manufacturing) and non-automatable (service) goods play an important role in the sectoral reallocation of labor, while the sectoral mobility of job seekers and the strength of the positive income effect versus the negative substitution effect on the demand for services due to a change in relative prices also matter for the extent of sectoral reallocation.

Finally, the model can replicate the magnitude of the decline in the ratio of manufacturing employment to service employment in Germany from 1994 to 2014. Specifically, we take from the German data the values of the capital share in manufacturing in these 2 years. Then, we compute the values of the degree of automation in our model that generate these two values in the corresponding steady states, keeping the rest of the calibration unchanged. We find that in the second steady state (for 2014) the model predicts a decline of $34\%$ in the ratio of manufacturing employment to service employment, which is close to the one found in the data ($30\%$). In addition, the model predicts a fall in the aggregate labor share of $7.7\%$, which matches well the data value ($7\%$).

Related Literature The paper brings together the strands of the literature on automation and structural change. To the best of our knowledge, we are the first to build a two-sector general equilibrium model with labor market frictions to analyze the long-run impact of automation on both sectoral and aggregate employment. Very few studies in the automation literature have considered a multi-sector economy but without accounting for labor frictions. Focusing on inequality, Berg et al. (2018) show that the inclusion of a non-automation sector amplifies the high-skill labor gains and low-skill labor losses from automation. In an overlapping generations setup with also a non-automatable sector, Sachs et al. (2019) study the possibility of one generation improving their welfare at future generations’ expense through robot adoption. The papers of Ngai and Pissarides (2008), Cruz and Raurich (2020) and Leon-Ledesma and Moro (2020) are examples of models that consider structural change with or without leisure (endogenous participation).^5^ Our contribution to the structural change literature is to investigate the effects of automation as a driver of sectoral reallocation in a search and matching framework.

In macroeconomic models with labor frictions, the role of automation remains little explored. Leduc and Liu (2020b) provide the first quantitative general equilibrium evaluation of the interaction between automation and labor market fluctuations over the business cycle. Automation acts as an endogenous wage rigidity by posing a threat to workers in wage negotiations. Leduc and Liu (2020a) extend this model with nominal rigidities. They find that pandemic-induced uncertainty shocks to worker productivity stimulate automation, which helps mitigate the negative impact on aggregate demand.^6^ We extend this literature by studying automation-driven sectoral reallocation.

Structure Section 2 lays out the model. Section 3 establishes the equilibrium relationship between relative labor demand and labor supply in the two-sector economy. Section 4 discusses the parameterization. Section 5 presents the results. Section 6 investigates the role of key parameters and features of the model. Section 7 concludes.

## The model

**Fig. 3:**
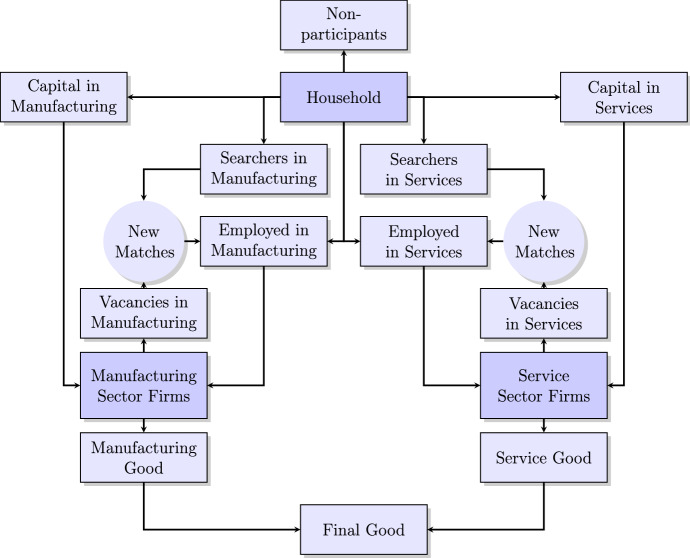
Model overview

We construct a general equilibrium model featuring search and matching frictions, endogenous labor decisions and two sectors (manufacturing and services). Figure 3 provides an overview of the model.

On the production side, there is a representative firm in each of the two sectors. Manufacturing output is produced with capital and labor as inputs. Automation increases the capital intensity of the technology in the manufacturing sector. This can be motivated by the idea that some work operations, formerly performed by humans, are now executed by robots (Acemoglu and Restrepo (2018)). Output in services is also produced with labor and capital. The outputs of the two sectors are costlessly aggregated into the final consumption good.

On the household side, there is a representative household consisting of employees, unemployed job seekers and labor force non-participants. The household rents out its capital to the manufacturing and service firms, purchases the final consumption good and receives dividends through owning the two firms.

### Labor markets

Jobs are created through a matching function. For $j=M,S$ denoting the manufacturing and service sectors, let $\upsilon _{t}^{j}$ be the number of vacancies and $u_{t}^{j}$ the number of job seekers. We assume matching functions of the form,

1 $$\begin{aligned} m_{t}^{j}=\mu _{1}^{j}(\upsilon _{t}^{j})^{\mu _{2}^{j}}(u_{t}^{j})^{1-\mu _{2}^{j}}, \end{aligned}$$

where the efficiency of the matching process is $\mu _{1}^{j}$ and $\mu _{2}^{j}$ denotes the elasticity of matches with respect to vacancies. For each sector, we define the hiring probability $\psi _{t}^{hj}$ and the vacancy-filling probability $\psi _{t}^{fj}$,

$$\begin{aligned} \psi _{t}^{hj}\equiv \frac{m_{t}^{j}}{u_{t}^{j}},\qquad \quad \psi _{t}^{fj}\equiv \frac{m_{t}^{j}}{\upsilon _{t}^{j}}. \end{aligned}$$

Labor market tightness $\theta _{t}^{j} \equiv v_{t}^{j}/u_{t}^{j}$ determines the matching market prospects of firms and workers. The probability that a worker finds a vacancy is an increasing function of labor market tightness, $\psi _{t}^{hj}=f(\theta _{t}^{j})$, while the probability that a job vacancy is matched with an unemployed worker is a decreasing function of tightness, $\psi _{t}^{fj}=f(\theta _{t}^{j})/\theta _{t}^{j}$.

In each period, jobs are destroyed at a constant fraction $\sigma ^{j}$ and $m_{t}^{j}$ new matches are formed. The law of motion of employment $n_{t}^{j}$ is then given by

2 $$\begin{aligned} n_{t+1}^{j}=(1-\sigma ^{j})n_{t}^{j}+m_{t}^{j}=(1-\sigma ^{j})n_{t}^{j}+\psi _{t}^{hj}u_{t}^{j} . \end{aligned}$$

Using the vacancy-filling probability, we obtain an equivalent expression,

3 $$\begin{aligned} n_{t+1}^{j}=(1-\sigma ^{j})n_{t}^{j}+\psi _{t}^{fj}\upsilon _{t}^{j}. \end{aligned}$$

### Household

Next, we present the structure of the household side in the model and the corresponding optimization problem.

#### Utility function and budget constraint

The representative household consists of a continuum of infinitely lived members. Utility is derived from consumption $$c_{t}$$ and from leisure, which corresponds to the fraction of members out of the labor force $$ l_{t}$$. The instantaneous utility function is given by

$$\begin{aligned} U(c_{t},l_{t})=\frac{c_{t}^{1-\eta }}{1-\eta }+\Phi \frac{l_{t}^{1-\varphi }}{1-\varphi }, \end{aligned}$$

where $\eta$ is the inverse of the intertemporal elasticity of substitution, $\Phi >0$ is the relative preference for leisure and $\varphi$ is the inverse of the Frisch elasticity of labor supply. At any point in time, a fraction $n_{t}^{M}$ ($n_{t}^{S}$) of the household’s members are employees in the manufacturing (service) sector. The household chooses the fraction of the unemployed actively searching for a job $u_{t}$ versus those who are out of the labor force enjoying leisure $l_{t}$ so that

4 $$\begin{aligned} n_{t}^{M}+n_{t}^{S}+u_{t}+l_{t}=1. \end{aligned}$$

Of the unemployed $u_t$, the household chooses the fraction of job seekers who look for a job in the manufacturing sector $s_t$ while the remaining $1-s_t$ search in services, so that

5 $$\begin{aligned} u_t = s_t u_t + (1-s_t)u_t = u_t^M + u_t^S, \end{aligned}$$

where $u_t^M \equiv s_t u_t$ and $u_t^S \equiv (1-s_t)u_t$. The household accumulates assets, evolving over time according to

6 $$\begin{aligned} k_{t+1}=i_{t}+(1-\delta )k_{t}, \end{aligned}$$

where $i_{t}$ is investment and $\delta$ is a constant depreciation rate. The household budget constraint is given by

7 $$\begin{aligned} c_{t}+i_{t} \le r_{t}k_{t} + w_{t}^{M}n_{t}^{M}+w_{t}^{S}n_{t}^{S} + {\bar{b}}_{t} u_{t} - T_{t} +\Pi _{t}^{M}+\Pi _{t}^{S}, \end{aligned}$$

where $w_{t}^{j}$ is the real wage in each sector, $r_{t}$ is the real return on assets, ${\bar{b}}_{t}$ is the unemployment benefit (see Sect. 4), $T_{t}$ refers to lump-sum taxes that adjust to satisfy the government budget, i.e., ${\bar{b}}_{t} u_{t} = T_{t}$ and $\Pi _{t}^{j}$ for $j=M,S$ denotes dividends received from ownership of the firms. We model the unemployment benefit as a share $\varpi$ of the average wage in the economy through the function ${\bar{b}}_{t} = \varpi \frac{(w^M_{t} n^M_{t} + w^S_{t} n^S_{t})}{n^M_{t}+n^S_{t}}$.

#### The optimization problem

The household maximizes the expected lifetime utility subject to Eqs. (1), (2), (4), (5), (6) and (7) (for details, see Online Appendix). Denoting by $\lambda ^{n^M}_t$, $\lambda ^{n^S}_t$ and $\lambda ^c_{t}$ the Lagrange multipliers on Eq. (2) for $j=S,M$ and (7), the first-order conditions with respect to $c_{t}$, $k_{t+1}$, $n_{t+1}^{M}$, $n_{t+1}^{S}$, $u_{t}$ and $s_t$ are given by

8 $$\begin{aligned}&c_t^{-\eta } = \lambda ^c_{t}, \end{aligned}$$

9 $$\begin{aligned}&\lambda ^c_{t} = \beta E_t \left[ \lambda ^c_{t+1} (1-\delta +r_{t+1}) \right] ,\end{aligned}$$

10 $$\begin{aligned}&\lambda ^{n^M}_t = \beta E_t \left[ -\Phi l_{t+1}^{-\varphi } + c_{t+1}^{-\eta } w_{t+1}^M + \lambda ^{n^M}_{t+1} (1-\sigma ^{M}) \right] , \end{aligned}$$

11 $$\begin{aligned}&\lambda ^{n^S}_t = \beta E_t \left[ -\Phi l_{t+1}^{-\varphi } + c_{t+1}^{-\eta } w_{t+1}^S + \lambda ^{n^S}_{t+1} (1-\sigma ^{S}) \right] , \end{aligned}$$

12 $$\begin{aligned}&\Phi l_{t}^{-\varphi } - \lambda ^{n^M}_t \psi _{t}^{h M} s_t-\lambda ^{n^S}_t \psi _{t}^{h S} (1-s_t) = \lambda ^c_{t} {\bar{b}}_{t}, \end{aligned}$$

13 $$\begin{aligned}&\lambda ^{n^M}_t \psi _t^{hM} = \lambda ^{n^S}_t \psi _t^{hS}. \end{aligned}$$

Equations (8) and (9) are the non-arbitrage conditions for the returns to consumption and capital. Equations (10) and (11) relate the expected marginal value of being employed in each sector to the utility loss from the reduction in leisure, the wage and the continuation value, which depends on the separation probability. Equation (12) states that the value of being unemployed (rather than enjoying leisure) should equal the marginal utility from leisure minus the expected marginal values of being employed in each sector, weighted by the respective job finding probabilities and shares of job seekers. Equation (13) states the choice of the share $s_t$ is such that the expected marginal values of being employed, weighted by the job finding probabilities, are equal in the two sectors. Notice that the marginal value to the household of an additional member employed in each sector is given by

14 $$\begin{aligned} V_{n^{M_t}}^{h}=-\Phi l_{t}^{-\varphi }+\lambda ^c_{t}w_{t}^{M}+(1-\sigma ^{M})\lambda ^{n^M}_t, \end{aligned}$$

15 $$\begin{aligned} V_{n^{S_t}}^{h}=-\Phi l_{t}^{-\varphi }+\lambda ^c_{t}w_{t}^{S}+(1-\sigma ^{S})\lambda ^{n^S}_t. \end{aligned}$$

### Production

We now turn to the structure of the production side in the economy and present the optimization problem of the firms in the two sectors.

#### Final good

There are three goods produced in the economy. These include two intermediate goods, namely manufacturing and service goods ($M_t$ and $S_t$), which are combined in the production of the final good $Y_t$ according to a CES technology,

16 $$\begin{aligned} Y_t = \left[ \gamma M_t^{\frac{\chi -1}{\chi }} + (1-\gamma ) S_t^{\frac{\chi -1}{\chi }} \right] ^{\frac{\chi }{\chi -1}}, \end{aligned}$$

where $0<\gamma <1$ denotes the weight attached to the manufacturing good versus the service good and $\chi$ is the elasticity of substitution.

The three goods are sold in competitive markets and we assume that the final good is the numeraire. Therefore, the prices of the sectoral goods equal the marginal products,

17 $$\begin{aligned} p_t^M= & {} \frac{\partial Y_t}{\partial M_t} = \gamma \left( \frac{ Y_t}{ M_t} \right) ^{\frac{1}{\chi }}, \end{aligned}$$

18 $$\begin{aligned} p_t^S= & {} \frac{\partial Y_t}{\partial S_t} = (1-\gamma ) \left( \frac{ Y_t}{ S_t} \right) ^{\frac{1}{\chi }}. \end{aligned}$$

#### Manufacturing intermediate good

The manufacturing good is produced by combining capital $k_t^M$ with employment $n_t^M$,

19 $$\begin{aligned} M_t = \left[ \zeta (k_t^M)^{\frac{\alpha -1}{\alpha }} + (1-\zeta ) (n_t^M )^{\frac{\alpha -1}{\alpha }} \right] ^{\frac{\alpha }{\alpha -1}}, \end{aligned}$$

where $\zeta$ denotes the weight attached to capital versus labor and $\alpha$ is the elasticity of substitution.

An increase in $\zeta$ makes output more capital-intensive at the expense of labor, representing in our setup an increased robot adoption (automation). The microeconomic foundations are derived by Acemoglu and Restrepo (2018) in a framework where a continuum of tasks is used in production. Automation in that context is interpreted as a shift in the share of tasks that can be produced with capital. Acemoglu and Restrepo (2018) show how one can aggregate the tasks to establish a production function with aggregate capital and labor inputs (see also the discussion in Bergholt et al. (2021)).

Firms maximize the discounted expected value of future profits subject to the technology and the law of motion of employment (2). That is, they take the number of workers currently employed $n_{t}^{j}$ as given and choose the number of vacancies to post $\upsilon _{t}^{j}$ so as to employ the desired number of workers next period $n_{t+1}^{j}$. The firm also chooses the amount of capital to demand. The manufacturing firm solves the problem

20 $$\begin{aligned} Q^M(n_{t}^{M})=\underset{\upsilon _{t}^{M}, k_t^M}{\max }\Big \{ p_{t}^{M} M_{t}-w_{t}^{M}n_{t}^{M} - r_t k_t^M -\kappa ^M \upsilon _{t}^{M} +E_{t}\left[ \Lambda _{t,t+1}Q^M(n_{t+1}^{M})\right] \Big \},\nonumber \\ \end{aligned}$$

where $\kappa ^M$ denotes the marginal cost of posting a vacancy. As the household owns the firm, the term $\Lambda _{t,t+1}=\beta \lambda ^c_{t+1}/\lambda ^c_{t}$ refers to the household’s stochastic discount factor in which $\lambda ^c_{t}$ denotes the Lagrange multiplier for the household budget constraint and $\beta$ is the household’s discount factor.

The first-order conditions with respect to $v_t^M$ and $k_t^M$ are

21 $$\begin{aligned} \kappa ^{M}= & {} \psi _{t}^{f M} \times E_{t} \Lambda _{t, t+1}\left[ p_{t+1}^{M} (1-\zeta ) \left( \frac{M_{t+1}}{n_{t+1}^M }\right) ^{\frac{1}{\alpha }}- w_{t+1}^{M}+ \frac{\left( 1-\sigma ^{M}\right) \kappa ^{M}}{\psi _{t+1}^{f M}}\right] ,\nonumber \\ \end{aligned}$$

22 $$\begin{aligned} r_t= & {} p_t^M \cdot \zeta \left( \frac{ M_t}{ k_t^M} \right) ^{\frac{1}{\alpha }}. \end{aligned}$$

Equation (21) states that the marginal cost of hiring a worker should equal the expected marginal benefit subject to the vacancy-filling probability. The latter includes the net value of the marginal product of labor, where $\zeta$ enters with a negative sign, minus the wage plus the continuation value. Equation (22) states that the return on capital is equal to the value of its marginal product, where $\zeta$ enters with a positive sign.

The value of the marginal job for the firm is given by

23 $$\begin{aligned} V_{n^{M}t}^{f}= p_{t}^{M} (1-\zeta ) \left( \frac{M_{t}}{n_{t}^M}\right) ^{\frac{1}{\alpha }}- w_{t}^{M}+ \frac{\left( 1-\sigma ^{M}\right) \kappa ^{M}}{\psi _{t}^{f M}}. \end{aligned}$$

#### Service intermediate good

In the service sector, the production function is given by

24 $$\begin{aligned} S_t = \left[ \xi (k_t^S)^{\frac{\rho -1}{\rho }} + (1-\xi ) (n_t^S )^{\frac{\rho -1}{\rho }} \right] ^{\frac{\rho }{\rho -1}}, \end{aligned}$$

where $\xi$ denotes the weight attached to capital versus labor and $\rho$ is the elasticity of substitution. A firm operating in this sector solves the following problem

25 $$\begin{aligned} Q^S(n_{t}^{S})=\underset{\upsilon _{t}^{S}, k_t^S}{\max }\Big \{ p_{t}^{S} S_{t}-w_{t}^{S}n_{t}^{S} - r_t k_t^S -\kappa ^S \upsilon _{t}^{S} +E_{t}\left[ \Lambda _{t,t+1}Q^S(n_{t+1}^{S})\right] \Big \},\quad \end{aligned}$$

The first-order conditions with respect to $v_t^S$ and $k_t^S$ are

26 $$\begin{aligned}&\displaystyle \kappa ^{S}=\psi _{t}^{f S} \times E_{t} \Lambda _{t, t+1}\left[ p_{t+1}^{S} (1-\xi ) \left( \frac{S_{t+1}}{n_{t+1}^S }\right) ^{\frac{1}{\rho }}- w_{t+1}^{S}+ \frac{\left( 1-\sigma ^{S}\right) \kappa ^{S}}{\psi _{t+1}^{f S}}\right] ,\nonumber \\ \end{aligned}$$

27 $$\begin{aligned}&\displaystyle r_t = p_t^S \cdot \xi \left( \frac{ S_t}{ k_t^S} \right) ^{\frac{1}{\rho }}. \end{aligned}$$

The value to the firm of a marginal job is given by

28 $$\begin{aligned} V_{n^{S}t}^{f}= p_{t}^{S} (1-\xi ) \left( \frac{S_{t}}{n_{t}^S}\right) ^{\frac{1}{\rho }}- w_{t}^{S}+ \frac{\left( 1-\sigma ^{S}\right) \kappa ^{S}}{\psi _{t}^{f S}}. \end{aligned}$$

### Wage bargaining

Following standard practice, the Nash bargaining problem in each sector is to maximize the weighted sum of log surpluses

29 $$\begin{aligned} \max _{w_{t}^{j}}\left\{ \left( 1-\vartheta ^{j}\right) \ln V_{n^{j} t}^{h}+\vartheta ^{j} \ln V_{n^{j} t}^{f}\right\} , \end{aligned}$$

where $$\vartheta ^{j}$$ denotes the bargaining power of firms and $$ V_{n^{j} t}^{h}$$, $$ V_{n^{j} t}^{f}$$ have been defined above. The first-order condition with respect to $$w_{t}^{j}$$ is

$$\begin{aligned} \vartheta ^{j} V_{n^{j} t}^{h}=\left( 1-\vartheta ^{j}\right) \lambda ^c_{t} V_{n^{j} t}^{f}. \end{aligned}$$

Through the derivations shown in Online Appendix, we obtain the equilibrium values for wages in the two sectors

30 $$\begin{aligned} w_{t}^{M}= & {} \left( 1-\vartheta ^{M}\right) \left( p_{t}^{M} (1-\zeta ) \left( \frac{M_{t}}{n_{t}^M}\right) ^{\frac{1}{\alpha }}+\frac{\left( 1-\sigma ^{M}\right) \kappa ^M}{\psi _{t}^{f M}}\right) \nonumber \\&+\frac{\vartheta ^{M}}{\lambda ^c_{t}}\left( \Phi l_{t}^{-\varphi }-\left( 1-\sigma ^{M}\right) \lambda ^{n^M}_t\right) , \end{aligned}$$

31 $$\begin{aligned} w_{t}^{S}= & {} \left( 1-\vartheta ^{S}\right) \left( p_{t}^{S} (1-\xi ) \left( \frac{S_{t}}{n_{t}^S}\right) ^{\frac{1}{\rho }}+\frac{\left( 1-\sigma ^{S}\right) \kappa ^S}{\psi _{t}^{f S}}\right) \nonumber \\&+ \frac{\vartheta ^{S}}{\lambda ^c_{t}}\left( \Phi l_{t}^{-\varphi }-\left( 1-\sigma ^{S}\right) \lambda ^{n^S}_t\right) . \end{aligned}$$

### Resource constraint

The final good is used for consumption and investment and also to cover vacancy costs.

32 $$\begin{aligned} Y_t = c_{t} + i_{t} + \kappa ^M \upsilon _{t}^{M} + \kappa ^S \upsilon _{t}^{S}. \end{aligned}$$

The derivation of the resource constraint is shown in Online Appendix.

## Relative labor demand and supply in the steady state

In this section, let us first provide the definition of steady-state equilibrium. We consider the long run as the interesting frequency given that the empirical counterpart of interest (Dauth et al. (2021)) focuses on long-run analysis, comparing the effects of automation in Germany between 1994 and 2014.

**Steady-state equilibrium** *A steady-state equilibrium is a set of values for prices* $\{p^M,p^S,r,w^M,w^S\}$ *and endogenous variables,* $\{u, v^M,v^S,k^S,k^M,s\}$, *such that*

1. *The law of motion of employment* (2) *holds in both sectors,*
2. *The prices of the intermediate sectoral goods,* $p^M$ *and* $p^S$, *equal the goods’ marginal products in the final good production,* *i.e.,* (17) *and* (18) *are satisfied,*
3. *The problem of the representative household is solved* (*Section* 2.2.2),
4. *The problem of the representative firm in each sector* (20*and* 25) *is solved,*
5. *Wages,* $w^M$ *and* $w^S$, *solve the respective bargaining problems* (29),
6. *The capital market clears, i.e.,* $k = k^M + k^S$.

Next, we establish the steady-state equilibrium relationship between relative labor demand and relative labor supply in the two sectors.

### Proposition 1

In the steady-state equilibrium, the sectoral ratio of labor market tightness depends only on the bargaining power and vacancy costs in the two sectors

$$\begin{aligned} \frac{\theta ^M}{\theta ^S} = \frac{\vartheta ^M}{(1-\vartheta ^M)}{\frac{(1-\vartheta ^S)}{\vartheta ^S}} \frac{\kappa ^S}{\kappa ^M}. \end{aligned}$$

### Proof

See Appendix. $$\square $$

Proposition 1 establishes that the *relative* labor market tightness of the two sectors is constant in the steady-state equilibrium and characterizes its level. Asymmetric bargaining power and/or vacancy costs introduce a wedge in tightness between the two sectors. Conversely, if both the bargaining power and vacancy costs are symmetric, tightness is equal in the two sectors. The derivation of Proposition 1 (see Appendix) builds on Ravn (2008), where a relationship between tightness and the marginal utility of consumption is derived in a one-sector search and matching model with endogenous participation.

The relationship between relative labor supply and relative labor demand directly follows from the proposition

$$\begin{aligned} \underbrace{\frac{s}{1-s}\equiv \frac{u^M}{u^S}}_{\begin{array}{c} \hbox {Relative labor}\\ \hbox {supply} \end{array}} = \frac{(1-\vartheta ^M)}{\vartheta ^M}{\frac{\vartheta ^S}{(1-\vartheta ^S)}} \frac{\kappa ^M}{\kappa ^S} \underbrace{\frac{v^M}{v^S}}_{\begin{array}{c} \hbox {Relative labor}\\ \hbox {demand} \end{array}}. \end{aligned}$$

For a given level of relative labor demand (which depends, among others, on the degree of automation $\zeta$), the pool of job seekers in manufacturing increases with the relative (i) bargaining power of workers and (ii) vacancy cost. In the second case, an increased pool of unemployed is required to compensate for the higher vacancy cost when firms decide about new vacancies so that the level of labor demand is sustained in equilibrium.

Finally, notice that the household decides how to allocate job seekers by comparing the discounted expected values of searching in the two sectors, $\psi ^{j,h} \beta E_t \left[ V^{h}_{n^{j_{t+1}}}\right]$, which, in turn, is equal to the probability of finding a job times the discounted expected value of being employed. The optimal value $s_t^*$ is given by

$$\begin{aligned} s_t^* = {\left\{ \begin{array}{ll} 1 &{}\quad \psi _t^{M,h} \beta E_t \left[ V^{h}_{n^{M_{t+1}}}\right] > \psi _t^{S,h} \beta E_t \left[ V^{h}_{n^{S_{t+1}}}\right] \\ s_t^* \in (0,1) &{}\quad \psi _t^{M,h} \beta E_t \left[ V^{h}_{n^{M_{t+1}}}\right] = \psi _t^{S,h} \beta E_t \left[ V^{h}_{n^{S_{t+1}}}\right] \\ 0 &{}\quad \psi _t^{M,h} \beta E_t \left[ V^{h}_{n^{M_{t+1}}}\right] < \psi _t^{S,h} \beta E_t \left[ V^{h}_{n^{S_{t+1}}}\right] . \\ \end{array}\right. } \end{aligned}$$

**Table 1:** Parameterization

Description		Value	Target/source
*Household*			
$\beta$	Discount factor	0.99	Return to capital, 5%
$\delta$	Depreciation rate	0.04	Standard calibration
$\Phi$	Relative utility from leisure	0.14	Participation Rate, 71%
$\phi$	Inverse Frisch elasticity of labor supply	2	Kneip et al. (2020)
$\eta$	Inverse elasticity of intertemporal substitution	2	Hansen and Singleton (1983)
*Production*			
$\chi$	Manufacturing–services elasticity of substitution	0.3	Ngai and Pissarides (2007)
$\gamma$	Share of manufacturing in total output	0.32	Sectoral output ratio, 0.891
$\zeta$	Weight attached to capital versus labor in manuf.	0.24	Capital share in manuf., 0.19
$\xi$	Weight attached to capital versus labor in services	0.36	Capital share in services, 0.28
$\alpha$, $\rho$	Capital–labor elasticities of substitution	0.8	Knoblach et al. (2020)
*Labor market*			
$\theta ^M$, $\theta ^S$	Bargaining power of firms	0.43, 0.6	Iftikhar and Zaharieva (2019)
$\mu _1$	Matching efficiency	0.58	Iftikhar and Zaharieva (2019)
$\mu _2$	Elasticity of matching to vacancies	0.46	Literature
$\sigma$	Separation rate	0.08	Iftikhar and Zaharieva (2019)
$\kappa$	Vacancy cost	0.11	Share of the average wage, 20%
$\varpi$	Replacement rate	0.6	OECD data

In the steady-state equilibrium, we can rule out the two corner solutions. If $s^* = 1$ and all the unemployed search in manufacturing, there is no production in services. Yet, as long as the two sectoral goods are not perfect substitutes in the final good production, the marginal product of the service good becomes infinite, leading to an infinite wage, which is incompatible with zero labor supply in this sector. If $s^*= 0$ and all the unemployed search in services, there is no production in manufacturing in the long run. Yet, as long as capital and labor are not perfect substitutes in manufacturing production, the marginal product of labor in manufacturing becomes infinite, which, again, is incompatible with a zero supply of labor in that sector. Therefore, the only possible solution is $s^* \in (0,1)$.

## Parameterization

In this section, we describe the calibration of the initial steady state, which we take to refer to the start year 1994 in the analysis of Dauth et al. (2021). We calibrate the model annually for the German economy. Some of the model parameters are taken from the literature. We choose the rest of the parameters to match a set of moments, using the simulated method of moments. Table 1 summarizes our parameterization.

Household We use the data set built by Jordà et al. (2019) to compute the return to capital *r* in Germany, which is equal to 5% in 1994. We set the capital depreciation rate $\delta$ equal to 4%. To choose the value for the discount factor, we use the Euler equation in the steady state, $\beta = 1 / (1+r-\delta )$. For the inverse elasticity of the intertemporal substitution $\eta$, much of the literature uses econometric estimates between 0 and 2 (see, e.g., Hansen and Singleton (1983)). The estimated aggregate Frisch elasticity for Germany varies between 0.85 and 1.06 in a micro panel of men in Germany from 2000 to 2013 used by Kneip et al. (2020). We thus set the Frisch elasticity to 0.85 ($\phi =2$). We have performed sensitivity analysis for different values $\phi =4,6$ (see Online Appendix and footnote 15). We calibrate the relative utility weight for leisure $\Phi$ to target a steady-state participation rate of $70\%$, in line with the data.

Production To calibrate the parameters of the aggregate production function, we set the share of manufacturing output $\gamma$ to 0.32 to match a sectoral output ratio of 0.891, measured by the ratio of value added in manufacturing and services in 1994. We set the elasticity of substitution between the two sectoral goods $\chi$ to 0.3, as in Ngai and Pissarides (2007). We set the weight attached to capital versus labor in manufacturing $\zeta$ by targeting the manufacturing capital share in 1994, which is equal to 0.19.^7^ Similarly, we set the value for the weight of capital in the production of services $\xi$ by targeting the capital share in the service sector in 1994, which is 0.28.^8^ We set the elasticity of substitution between capital and labor in manufacturing and services, $\alpha$ and $\rho$, equal to 0.6. Based on a meta-regression sample, Knoblach et al. (2020) estimate a long-run elasticity for the aggregate economy in the range of 0.45–0.87, noting that most industrial estimates do not deviate significantly from the estimate for the aggregate economy. Oberfield and Raval (2020) find the US manufacturing sector’s aggregate elasticity to be in the range of 0.5–0.7.

**Labor markets** To calibrate the parameters for the bargaining power of firms in each sector, we take weighted averages of the estimates for high-skill and low-skill workers in Iftikhar and Zaharieva (2019). A lower bargaining power for workers in the service sector is in line with the empirical evidence that service workers get a lower fraction of output produced in their sector, leading to a mild wage premium in manufacturing of around 2% in our calibration. The same authors estimate the average job duration rate in Germany to be 12.25 years, so we set the destruction rate in both sectors as $\sigma = 1/12.25 = 0.08$. We set the gross replacement rate $\varpi$ equal to 0.6.^9^ For the vacancy cost parameter, we set in both sectors $\kappa =0.1$, which implies that vacancy costs represent around 20% of the average wage. We set the matching efficiency parameter $\mu _1$ equal to 0.58, in line with the estimate in Iftikhar and Zaharieva (2019). We also perform sensitivity analysis for $\mu _1=0.4,0.5$ (see Online Appendix). We set the elasticity of the matching function with respect to vacancies $\mu _2$ equal to 0.46. This value is close to 0.5, often assumed in the search and matching literature, and also close to the estimate of 0.54 in Iftikhar and Zaharieva (2019), based on aggregate data of the Federal Employment Agency.

## Automation and sectoral reallocation: long-run analysis

In this section, we present the main results of our quantitative analysis.

### Steady-state results (untargeted moments)

Let us first report three side statistics to get an idea of the overall performance of our quantitative theory. In Table 2, we report the steady-state aggregate labor share, the aggregate unemployment rate and the sectoral employment ratio. The overall picture that emerges shows that our model does a good job in providing satisfactory values for these side statistics.

**Table 2:** Steady-state results (untargeted moments)

Variable	Expression	Model	Data
Labor share: aggregate	$\frac{w^Mn^M+w^Sn^S}{Y}$	0.72	0.76
Unemployment rate	$\frac{u}{1-l}$	0.09	0.08
Labor ratio: manuf./services	$\frac{n^M}{n^S}$	0.99	0.86

### Analysis across steady states

**Fig. 4:**
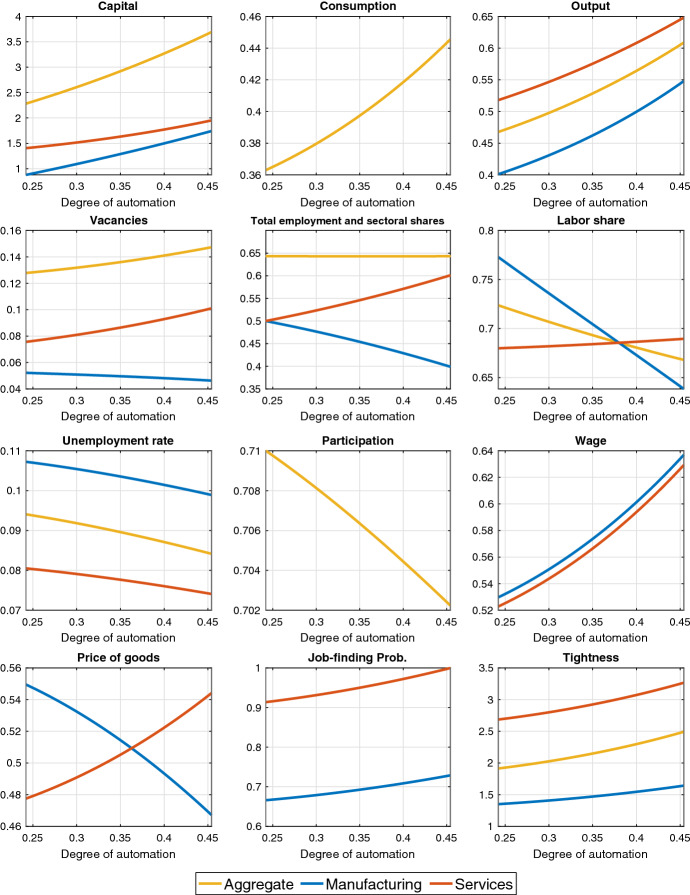
Steady-state effects of automation in a two-sector economy. *Note* The y-axis shows steady-state levels

Next, we discuss steady-state comparative statics with respect to an increase in the degree of automation $\zeta$. Figure 4 depicts the results for the main variables in the model for $0.24<\zeta <0.45$. The lower limit for $\zeta$ is the same as in Table 1. The upper limit for $\zeta$ is chosen by targeting a manufacturing capital share of 0.34 in 2014, which is the end year in the empirical analysis of Dauth et al. (2021).

Sectoral reallocation of output A higher degree of automation $\zeta$ corresponds to an increased (decreased) capital (labor) intensity of manufacturing production. Therefore, an increase in $\zeta$ reduces the importance of the limiting factor, labor, in the production of the manufacturing good and the capital demand of the manufacturing sector increases. Since the steady-state return to capital is constant, while the steady-state return to labor can freely adjust, the capital increase due to a higher $\zeta$ dominates the labor decline. Therefore, manufacturing output increases.^10^ Also, the level of output in services increases. Therefore, the economy experiences an aggregate output expansion. Overall, a higher $\zeta$ increases the steady-state ratio of manufacturing to service output *M*/*S* and decreases the relative price of the manufacturing good (see Eqs. (17) and (18)).

**Consumption, participation and labor share** The positive effect on aggregate income explains the increase in consumption and the decrease in participation in the long run. Automation has a negative effect on the aggregate labor income share, which is driven by the manufacturing sector and is in line with the previous evidence from the literature on the importance of the automation mechanism for a countercyclical labor share (see, e.g., Bergholt et al. (2021) and Leduc and Liu (2020b)).

**Sectoral reallocation of labor** Vacancies in the manufacturing sector decrease. Automation affects labor demand in manufacturing through two competing channels: (a) production becomes less labor-intensive, which tends to decrease employment *(labor-intensity channel)* and (b) since capital and labor are complements, the increase in capital tends to increase labor demand *(capital–labor complementarity effect)*. Vacancies in services increase due to the expansion in the demand for services. Total vacancies increase as well.

**Fig. 5:**
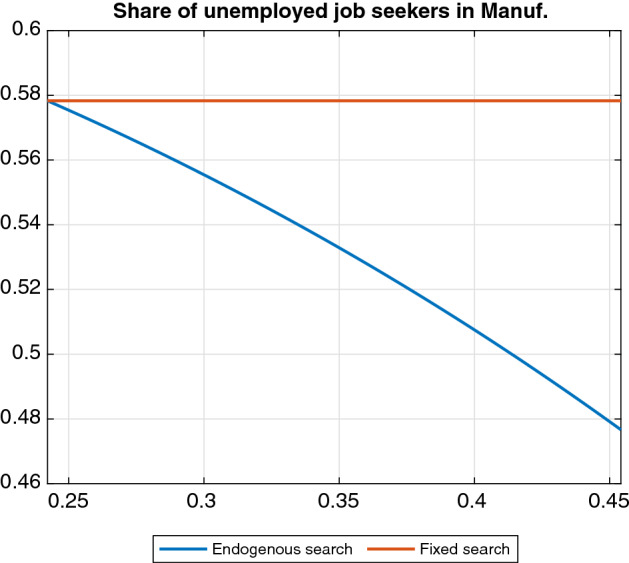
Steady-state effect of automation on searchers’ share in manufacturing. *Note* The y-axis shows steady-state levels. The blue line refers to the baseline model, whereas the red line refers to a model variant where the sectoral allocation of job seekers is kept fixed

The number of unemployed searchers drops in the manufacturing sector as households reduce participation and reallocate job search toward services. The unemployment rate drops in the service sector too, but the share of searchers increases (see blue line in Figure 5). Total unemployment falls.

Labor market tightness increases in both sectors. The effect on the hiring rates follows from the fact that they are a positive function of tightness (while the opposite holds for vacancy-filling rates). The impact of automation on wages in both sectors is positive, consistently with the decrease in the vacancy-filling probabilities.

Following the sectoral reallocation of labor, employment increases in services and falls in manufacturing in such a way that aggregate employment remains relatively constant, in line with the empirical evidence in Dauth et al. (2021). The pattern matches well the one observed in Fig. 1.

In sum, labor markets adjust to automation through vacancy creation, sectoral reallocation of the unemployed, and participation. The findings also highlight the expansionary effects of automation on the economy, namely the aggregate output expansion and unemployment reduction.

### Reproducing the size of the shift in key variables

To assess how well our model can explain the sectoral reallocation of employment in Germany, we focus next on comparing two steady states in Table 3, namely with $\zeta =0.24$ (targeting a manufacturing capital share equal to 0.19 in 1994) and $\zeta =0.45$ (targeting a manufacturing capital share equal to 0.34 in 2014).

Let us first examine the steady-state values for the ratio of manufacturing employment to service employment for these two values of $\zeta$. The model predicts a decline of $34\%$ in the ratio of manufacturing employment to service employment, which is reasonably close to the one found in the aggregate data for the German economy ($30\%$). Turning next to the aggregate labor share, the model predicts a fall of $7.7\%$, which is extremely close to the value in the data ($7\%$). For the labor share in manufacturing, the model predicts a decline of $17.4\%$, which again matches well with the observed change in the data ($18\%$). Finally, for the labor share in services, the model predicts a small increase of $1.4\%$, while in the data the change is essentially zero.

Overall, the model can reproduce satisfactorily the magnitude of the decline in the labor share and in the ratio of manufacturing employment to service employment in Germany between 1994 and 2014.

**Table 3:** Changes between two steady states and model fit to data

Variable	Expression	Steady	Steady	Change:	Change:
		State 1	State 2	Model	Data
Degree of automation	$\zeta$	0.24	0.45	88%	N/A
Manuf. capital share	$\frac{rK^M}{p^M M}$	0.19	0.34	71%	71%
Sectoral labor ratio	$\frac{n^M}{n^S}$	0.99	0.66	−34%	−30%
Labor share: aggregate	$\frac{w^Mn^M+w^Sn^S}{Y}$	0.72	0.67	−7.7%	−7%
Labor share: services	$\frac{w^Sn^S}{p^SS}$	0.68	0.69	1.4%	0%
Labor share: manuf.	$\frac{w^Mn^M}{p^MM}$	0.77	0.64	−17.4%	−18%

*Note* In steady state 1 and steady state 2, the degree of automation $\zeta$ *is set to target the capital share in German manufacturing in 1994 and 2014, respectively. The change in the manufacturing capital share in the model and data is therefore the same by construction*

**Fig. 6:**
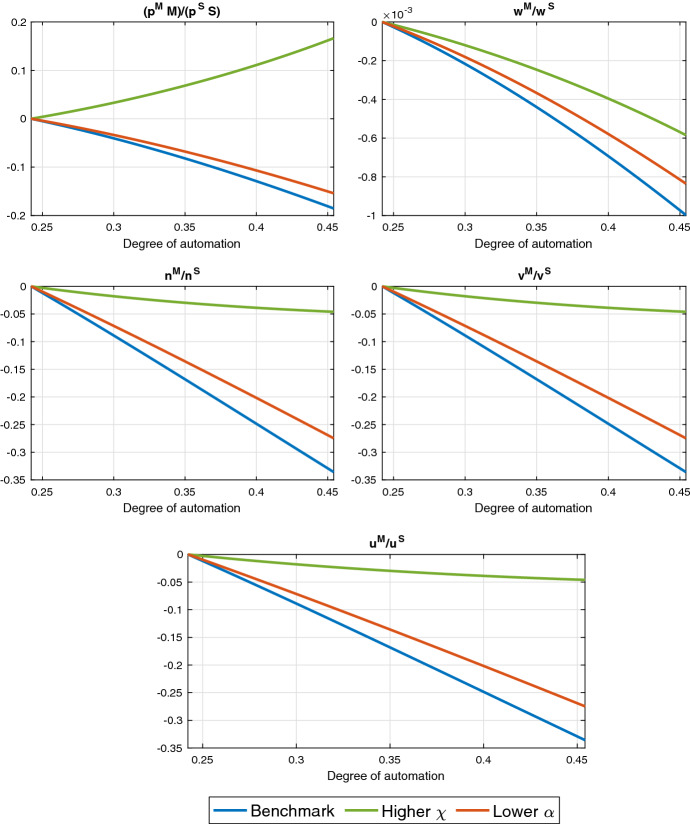
Steady-state effects of automation in a two-sector economy: Different elasticities of substitution between capital and labor ($\alpha = 0.7$) and between the two sectoral goods ($\chi = 1.5$) *Note: All the plotted variables are normalized to zero in the initial steady state. We denote the ratios of manufacturing to services variables as follows:* $p^MM/p^SS$ *for the value of output,* $w^M/w^S$ *for wages,* $n^M/n^S$ *for labor,* $v^M/v^S$ *for vacancies and* $u^M/u^S$ *for job seekers*

## What determines the extent of sectoral reallocation?

In this section, we investigate the role of key parameters and features of the model, namely (i) the elasticity of substitution between the sectoral goods, (ii) the elasticity of substitution between capital and labor in the automatable sector and (iii) the sectoral mobility of job seekers.

### Elasticities of substitution

Between the Sectoral Goods The elasticity of substitution between the sectoral goods $\chi$ matters for the sectoral reallocation of output and labor. Figure 6 compares the change in key sectoral ratios of variables as the degree of automation $\zeta$ increases from the initial steady state (with $\zeta = 0.24$) for a higher elasticity $\chi$ and for our benchmark calibration. Additional variables and the same results in levels of these ratios are included in Online Appendix. Relative to the baseline calibration ($\chi =0.3$), when we increase the elasticity ($\chi = 1.5$), the sectoral output ratio *M*/*S* changes by more due to automation because it is easier now to substitute services by manufacturing intermediate goods in the final good production. Even when manufacturing and services are gross substitutes ($\chi = 1.5$), output in services increases.^11^ This is because of two different effects that have the same sign in our baseline calibration and opposite signs when we increase $\chi$.

Firstly, the changes in the demand for services and manufacturing goods are affected by the standard income and substitution effects due to a change in the relative price of the manufacturing good. On the one hand, the increase in automation and the accumulation of capital leads to a decrease in the marginal cost of production in manufacturing, given the constant rental rate of capital in steady state. That is, the relative price of the manufacturing good relative to services in the production of the final good decreases.^12^ This implies a negative substitution effect on the use of services in the final good production. On the other hand, the reduction in the cost of manufacturing has a positive income effect for both inputs in the final good sector. In our baseline calibration, the positive income effect dominates, while with higher $\chi$ the negative substitution effect dominates, as can be seen in the evolution of the expenditure ratio for manufacturing and service goods in the upper left panel of Fig. 6.

Secondly, the increase in the capital stock (which represents household wealth) due to automation in the long run generates a positive wealth effect that increases the demand for services and manufacturing goods. This second effect leads to an increase in service production for both calibrations, despite the fact that manufacturing and services are gross substitutes if $\chi >1$.

However, the stronger substitution effect reduces the degree of sectoral reallocation if the elasticity of substitution is higher, despite an overall increase in service production. Consequently, an increase in $\chi$ mitigates the effect of automation on the sectoral reallocation of output, labor, vacancies and job seekers (see the plots of the sectoral labor ratios $n^M/n^S$, $v^M/v^S$, and $u^M/u^S$). In line with these results, the drop in the wage premium in manufacturing $w^M/w^S$ becomes less pronounced and total employment decreases.^13^

Between Capital and Labor in the Automatable Sector The elasticity of substitution between capital and labor in manufacturing matters for the sectoral reallocation of labor. Figure 6 also depicts results for a lower value of this elasticity ($$\alpha = 0.7$$). Through the capital–labor complementarity channel, a decrease in $$\alpha $$ tends to dampen the automation-driven sectoral reallocation of vacancies, job seekers, and labor as well as the drop in the wage premium in manufacturing (see the plots of the sectoral labor ratios $$v^M/v^S$$, $$u^M/u^S$$, $$n^M/n^S$$, and $$w^M/w^S)$$.

### Sectoral mobility of job seekers

Last, we explore the extent to which shutting down the reallocation of job seekers between the two sectors affects our findings. We examine the comparative statics wth (a) endogenous sector-specific search (as in the baseline model) and (b) fixed sectoral shares of job seekers by keeping the share of searchers in manufacturing *s* equal to the value it attains endogenously in the initial steady state with ${\zeta =0.24}$ (see Fig. 5). In other words, Eq. (13) is no longer used. Hence, although the number of employees per sector can evolve separately through the dynamics of vacancy postings, matches and participation, households cannot freely reallocate job seekers between sectors.

**Fig. 7:**
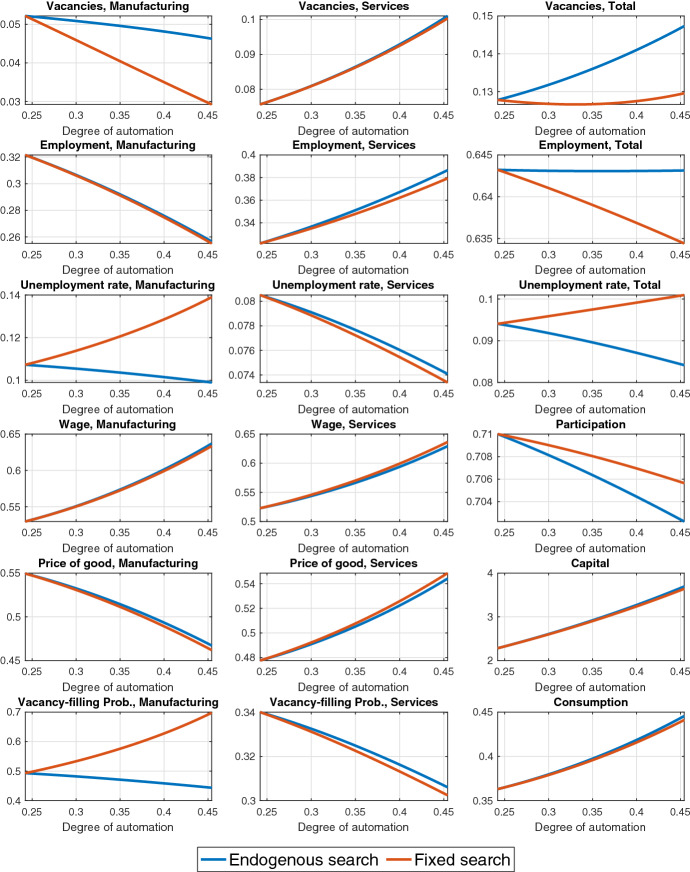
Steady-state effects of automation with and without sectoral mobility. *Note: The y-axis shows steady-state levels. The blue line refers to the baseline model, whereas the red line refers to a model variant where the sectoral allocation of job seekers is kept fixed*

With a fixed sectoral allocation of job seekers, as we move from a steady state with ${\zeta =0.24}$ to a steady state with $\zeta =0.45$ (in line with Table 3), total employment decreases, rather than remaining constant as with endogenous allocation (see Fig. 7).^14^ If job seekers cannot switch sector, the unemployment rate in manufacturing increases with ${\zeta }$, driving an increase in total unemployment. At the same time, the decrease in the unemployment rate in services becomes sharper since there is less job competition in this market without the sectoral reallocation of job seekers. Finally, the sectoral mobility of job seekers also matters for the effect of automation on manufacturing vacancies with the decline becoming stronger under fixed search. The positive wealth effect for the household (increase in consumption and decrease in participation) is weakened under fixed search.^15^

## Conclusion

The paper studies the sectoral impact of automation through the lens of a general equilibrium model with matching frictions, endogenous participation and two sectors. As in empirical evidence from Germany (see Dauth et al. (2021)), automation induces firms to create fewer new vacancies and job seekers to search less in the robot-exposed sector. Analysis across steady states shows that the reduction in manufacturing employment from automation can be offset by the increased service employment, thus leaving aggregate employment unaffected. The model does a good job in replicating (a) qualitatively the empirical evolution of employment and labor compensation in manufacturing and services and (b) the magnitude of the decline in the aggregate labor share and the ratio of manufacturing employment to service employment between 1994 and 2014.

Our model can be extended along several dimensions. For instance, the good produced in the automated sector (manufacturing) is, in fact, a tradable good. One plausible extension could therefore be to consider the sectoral impact of automation in an open economy framework. Another interesting avenue for further research would be to introduce skill heterogeneity and capital-skill complementarity (see, e.g., Dolado et al. (2021), Santini (2021)). Such a setup could capture the idea that robots are complements with high-skill workers but substitutes for low-skill workers, allowing to study implications for inequality. We leave these topics for future research.

### Supplementary Information

Below is the link to the electronic supplementary material.

Supplementary material 1 (pdf 618 KB)

## Appendix: Proof of Proposition 1

### Proof

From the maximization problem of the household, we have

A.1 $$\begin{aligned} \Phi l_{t}^{-\varphi }=\lambda ^{n^M}_t \psi _{t}^{h M} s_{t}+\lambda ^{n^S}_t \psi _{t}^{h S}\left( 1-s_{t}\right) +\lambda ^c_{t} {\bar{b}}_t, \end{aligned}$$

and

A.2 $$\begin{aligned} \lambda ^{n^M}_t \psi _{t}^{h M}=\lambda ^{n^S}_t \psi _{t}^{h S}. \end{aligned}$$

We can substitute (A.2) into (A.1) and obtain

$$\begin{aligned} \Phi l_{t}^{-\varphi }=\lambda ^{n^S}_t \psi _{t}^{h M} +\lambda ^c_{t} {\bar{b}}_t, \end{aligned}$$

or alternatively we can get,

$$\begin{aligned} \Phi l_{t}^{-\varphi }=\lambda ^{n^S}_t \psi _{t}^{h S} +\lambda ^c_{t} {\bar{b}}_t, \end{aligned}$$

which states that the marginal utility of leisure is equal to the value of being unemployed. The latter in turn is equal to the utility value of the unemployment benefit plus the probability of finding a job times the value of being employed. We invert these equations and obtain

$$\begin{aligned} \lambda ^{n^M}_t =\frac{\Phi l_{t}^{-\varphi } - \lambda ^c_{t} {\bar{b}}_t}{ \psi _{t}^{h M}}, \end{aligned}$$

and

$$\begin{aligned} \lambda ^{n^S}_t = \frac{\Phi l_{t}^{-\varphi } - \lambda ^c_{t} {\bar{b}}_t}{\psi _{t}^{h S}}. \end{aligned}$$

The values of an additional unit of employment in the two sectors are

$$\begin{aligned} V_{n^{M_{t}}}^{h}=\lambda ^c_{t} w_{t}^{M}-\Phi l_{t}^{-\varphi }+\left( 1-\sigma ^{M}\right) \lambda ^{n^M}_t, \end{aligned}$$

and

$$\begin{aligned} V_{n^{S_{t}}}^{h}=\lambda ^c_{t} w_{t}^{S}-\Phi l_{t}^{-\varphi }+\left( 1-\sigma ^{S}\right) \lambda ^{n^S}_t. \end{aligned}$$

The Lagrange multipliers $\lambda ^{n^M}_t$ and $\lambda ^{n^S}_t$ are equal to

$$\begin{aligned} \lambda ^{n^M}_t=\beta E_{t}\left[ \lambda ^c_{t+1} w_{t+1}^{M}-\Phi l_{t+1}^{-\varphi }+\lambda ^{n^M}_{t+1}(1-\sigma ^M)\right] , \end{aligned}$$

and

$$\begin{aligned} \lambda ^{n^S}_t=\beta E_{t}\left[ \lambda ^c_{t+1} w_{t+1}^{S}-\Phi l_{t+1}^{-\varphi }+\lambda ^{n^S}_{t+1}(1-\sigma ^S)\right] . \end{aligned}$$

Therefore, we can write

A.3 $$\begin{aligned} \lambda ^{n^S}_t=\beta E_{t}\left[ V_{n^{S_{t+1}}}^{h} \right] , \end{aligned}$$

and

A.4 $$\begin{aligned} \lambda ^{n^M}_t=\beta E_{t}\left[ V_{n^{M_{t+1}}}^{h} \right] . \end{aligned}$$

Consider now the problems of the two representative firms where the first-order conditions with respect to vacancies are given by

$$\begin{aligned} \frac{\kappa ^{M}}{\psi _{t}^{f M}}=E_{t} \Lambda _{t, t+1}\left[ p_{t+1}^{M}(1-\zeta )\left( \frac{M_{t+1}}{n_{t+1}^{M}}\right) ^{\frac{1}{\alpha }}-w_{t+1}^{M}+\frac{\left( 1-\sigma ^{M}\right) \kappa ^{M}}{\psi _{t+1}^{f M}}\right] , \end{aligned}$$

and

$$\begin{aligned} \frac{\kappa ^{S}}{\psi _{t}^{f S}}=E_{t} \Lambda _{t, t+1}\left[ p_{t+1}^{S} (1-\xi ) \left( \frac{S_{t+1}}{n_{t+1}^S }\right) ^{\frac{1}{\rho }}- w_{t+1}^{S}+ \frac{\left( 1-\sigma ^{S}\right) \kappa ^{S}}{\psi _{t+1}^{f S}}\right] . \end{aligned}$$

The marginal value of an extra unit of employment in period *t* for each sector is

$$\begin{aligned} V_{n^{M} t}^{f}=p_{t}^{M}(1-\zeta )\left( \frac{M_{t}}{n_{t}^{M}}\right) ^{\frac{1}{\alpha }}-w_{t}^{M}+\frac{\left( 1-\sigma ^{M}\right) \kappa ^{M}}{\psi _{t}^{f M}}, \end{aligned}$$

and

$$\begin{aligned} V_{n^{S}t}^{f}= p_{t}^{S} (1-\xi ) \left( \frac{S_{t}}{n_{t}^S}\right) ^{\frac{1}{\rho }}- w_{t}^{S}+ \frac{\left( 1-\sigma ^{S}\right) \kappa ^{S}}{\psi _{t}^{f S}}. \end{aligned}$$

Therefore, we can write

A.5 $$\begin{aligned} \frac{\kappa ^{M}}{\psi _{t}^{f M}}=E_{t} \Lambda _{t, t+1}\left[ V_{n^{M} t+1}^{f}\right] , \end{aligned}$$

and

$$\begin{aligned} \frac{\kappa ^{S}}{\psi _{t}^{f S}}=E_{t} \Lambda _{t, t+1}\left[ V_{n^{S} t+1}^{f}\right] . \end{aligned}$$

Recall that the first-order conditions of the wage bargaining problems are

A.6 $$\begin{aligned} \vartheta ^{M} V_{n^{M} t}^{h}=\left( 1-\vartheta ^{M}\right) \lambda ^c_{t} V_{n^{M} t}^{f}, \end{aligned}$$

and

$$\begin{aligned} \vartheta ^{S} V_{n^{S} t}^{h}=\left( 1-\vartheta ^{S}\right) \lambda ^c_{t} V_{n^{S} t}^{f}. \end{aligned}$$

By evaluating Eq. (A.6) for the next period, multiplying by $\frac{\beta }{\lambda ^c_{t}}$ and taking expectations, we obtain

$$\begin{aligned} \frac{\vartheta ^{M}}{\lambda ^c_{t}} \beta E_t \left[ V_{n^{M} t+1}^{h}\right] =\left( 1-\vartheta ^{M}\right) E_t \Lambda _{t, t+1} \left[ V_{n^{M} t+1}^{f}\right] . \end{aligned}$$

Substituting (A.4) and (A.5), we get

$$\begin{aligned} \frac{\vartheta ^{M}}{\lambda ^c_{t}} \frac{\left( \Phi l_{t}^{-\varphi } - \lambda ^c_{t} {\bar{b}}_t\right) }{ \psi _{t}^{h M}}=\left( 1-\vartheta ^{M}\right) \frac{\kappa ^{M}}{\psi _{t}^{f M}}, \end{aligned}$$

and, after rearranging terms, we obtain

$$\begin{aligned} \theta _t^M = \frac{\vartheta ^M}{1-\vartheta ^M} \frac{\left( \Phi l_{t}^{-\varphi } - \lambda ^c_{t} {\bar{b}}_t\right) }{\kappa ^M}. \end{aligned}$$

Similarly for the service sector, we have

$$\begin{aligned} \theta _t^S = \frac{\vartheta ^S}{1-\vartheta ^S} \frac{\left( \Phi l_{t}^{-\varphi } - \lambda ^c_{t} {\bar{b}}_t\right) }{\kappa ^S}. \end{aligned}$$

These relations are similar to the linear relationship between labor market tightness and the marginal utility of consumption derived by Ravn (2008) in a one-sector search and matching model with endogenous participation. By taking the ratio of tightness in the two sectors and considering the steady-state equilibrium, we obtain the relationship of Proposition 1.

$$\begin{aligned} \frac{\theta ^M}{\theta ^S} = \frac{\frac{\vartheta ^M}{1-\vartheta ^M}}{\frac{\vartheta _S}{1-\vartheta _S}} \cdot \frac{\kappa ^S}{\kappa ^M}. \end{aligned}$$

$$\square $$

## Footnotes

1 There are two main strands in the literature regarding a tangible measure of automation: information and communication technology capital (see, e.g., Eden and Gaggl (2018)) and robotics (see, e.g., Graetz and Michaels (2018)).

2 As one of the most important manufacturing exporters worldwide, Germany is a special case. Therefore, the results in this paper cannot be generalized to other economies without further research.

3 For empirical work on the decline in manufacturing and the rise in services, see a novel dataset for 10 sectors, 23 countries and 150 years compiled by Priftis and Shakhnov (2020).

4 Bergholt et al. (2021) examine impulse responses to an automation shock, modeled as an exogenous increase in the weight of capital in the production function of a New Keynesian model. They find that automation is the main driver of the long-run labor share.

5 See also the survey by Herrendorf et al. (2014) and the model of structural change, skills mismatch and matching frictions in Restrepo (2015).

6 Models with automation, heterogeneous households and matching frictions are developed by Cords and Prettner (2019) and Jaimovich et al. (2020) to study the impact on inequality.

7 EUKLEMS defines the capital share as the ratio of capital services to value added. Following Iftikhar and Zaharieva (2019), we define our manufacturing sector as the aggregate of Industries A-F in the German WZ08 industry classification.

8 We calculate this value using EUKLEMS data for industries that are defined as “Market Economy,” excluding the set of industries (A-F) that define our automatable sector (manufacturing).

9 According to the OECD, the standard rates in Germany after 2000 are 60% of the previous earnings net of tax.

10 The effect of an increase in $\zeta$ on manufacturing output *M* is expressed by the derivative,

$$\begin{aligned} \frac{\partial M}{\partial \zeta } = \frac{1}{\alpha } M^{(1-\alpha )} \left[ (k^M)^\alpha - (n^M)^\alpha + \zeta \alpha \frac{\partial k}{\partial \zeta } + (1-\zeta ) \alpha \frac{\partial n^M}{\partial \zeta } \right] . \end{aligned}$$

An increase in $\zeta$ induces an accumulation of capital $(\frac{\partial k^M}{\partial \zeta }>0)$ in the long run and a decrease in employment $(\frac{\partial n^M}{\partial \zeta }<0)$. The difference $(k^M)^\alpha - (n^M)^\alpha$ also matters for which effect dominates. If the initial value of $\zeta$ is sufficiently low, the steady-state capital stock $k^M$ is relatively low and labor $n^M$ is relatively more important in the production, leading to a decrease in manufacturing output.

11 See the upper middle panel of Figure A.1 in Online Appendix.

12 We show an empirical counterpart of the relative prices for manufacturing and services in Figure A.6 of Online Appendix.

13 See Figure A.1 in Online Appendix.

14 Figure 7 omits the output and labor share variables as the differences between the two model variants are minimal. Results are available upon request.

15 In Online Appendix, we also show results for different values of the parameter governing the Frisch elasticity of labor supply ($\phi =4,6$). A lower value of the Frisch elasticity (higher value of $\phi$) matters for the steady-state levels of the variables but without affecting our main results.
