# Supplementary material for: Automation and sectoral reallocation
Source: SERIEs (Berl). 2021 Jul 30;13(1-2):335–62. doi: 10.1007/s13209-021-00240-w (PMC8323451; doi:10.1007/s13209-021-00240-w)
Supplement: Supplementary file 1 — Supplementary material 1 (pdf 618 KB) [file 13209_2021_240_MOESM1_ESM.pdf]

Automation and Sectoral Reallocation

Online Appendix

Dennis C. Hutschenreiter[1] Tommaso Santini[2]

Eugenia Vella[3]

June 12, 2021

[1]Universitat Autònoma de Barcelona and Barcelona GSE. *e-mail:* Dennis.Hutschenreiter@uab.cat

[2]Universitat Autònoma de Barcelona and Barcelona GSE. *e-mail:* tommaso.santini@uab.cat.

[3]Athens University of Economics and Business and Fundació MOVE. *e-mail:* evella@aueb.gr

1 The maximization problem of the household

The Lagrangian is given by:

$$\begin{aligned}\mathcal{L} = E_0 \sum_{t=0}^{\infty} \beta^t \Bigg\{ & \frac{c_t^{1-\eta}}{1-\eta} + \Phi \frac{\left[1 - u_t - n_t^M - n_t^S\right]^{1-\varphi}}{1-\varphi} \\ & - \lambda_t^c \left[c_t + k_{t+1} - (1-\delta)k_t - r_t k_t - w_t^M n_t^M - w_t^S n_t^S - \bar{b}_t u_t - \Pi_t^M - \Pi_t^S\right] \\ & - \lambda_t^{n^M} \left[n_{t+1}^M - \left(1 - \sigma^M\right) n_t^M - \psi_t^{hM} s_t u_t\right] \\ & - \lambda_t^{n^S} \left[n_{t+1}^S - \left(1 - \sigma^S\right) n_t^S - \psi_t^{hS} (1 - s_t) u_t\right] \Bigg\}.\end{aligned}$$

The first-order conditions with respect to c_t, k_{t+1}, n_{t+1}^M, n_{t+1}^S, u_t, and s_t are,
[wrt c_t]

$$c_t^{-\eta} - \lambda_t^c = 0, \tag{A.1}$$

[wrt k_{t+1}]

$$- \beta^t \lambda_t^c + \beta^{t+1} E_t \left[\lambda_{t+1}^c (1 - \delta + r_{t+1})\right] = 0, \tag{A.2}$$

[wrt n_{t+1}^M]

$$- \beta^t \lambda_t^{n^M} + \beta^{t+1} E_t \left[-\Phi l_{t+1}^{-\varphi} + \lambda_{t+1}^c w_{t+1}^M + \lambda_{t+1}^{n^M} (1 - \sigma^M)\right] = 0, \tag{A.3}$$

[wrt n_{t+1}^S]

$$- \beta^t \lambda_t^{n^S} + \beta^{t+1} E_t \left[-\Phi l_{t+1}^{-\varphi} + \lambda_{t+1}^c w_{t+1}^S + \lambda_{t+1}^{n^S} (1 - \sigma^S)\right] = 0, \tag{A.4}$$

[wrt u_t]

$$- \beta^t \Phi l_t^{-\varphi} + \beta^t \lambda_t^c \bar{b}_t + \beta^t \lambda_t^{n^M} \psi_t^{hM} s_t + \beta^t \lambda_t^{n^S} \psi_t^{hS} (1 - s_t) = 0, \tag{A.5}$$

[wrt s_t]

$$\lambda_t^{n^M} \psi_t^{hM} - \lambda_t^{n^S} \psi_t^{hS} = 0. \tag{A.6}$$

To simplify the system of equations, we combine (A.1) and (A.2),

$$c_t^{-\eta} = \beta E_t \left[c_{t+1}^{-\eta} (1 - \delta + r_{t+1})\right], \tag{A.7}$$

and then rearrange terms in (A.3) - (A.6),

$$\lambda_t^{n^M} = \beta E_t \left[-\Phi l_{t+1}^{-\varphi} + c_{t+1}^{-\eta} w_{t+1}^M + \lambda_{t+1}^{n^M} (1 - \sigma^M)\right], \tag{A.8}$$

$$\lambda_t^{n^S} = \beta E_t \left[-\Phi l_{t+1}^{-\varphi} + c_{t+1}^{-\eta} w_{t+1}^S + \lambda_{t+1}^{n^S} (1 - \sigma^S)\right], \tag{A.9}$$

$$\Phi l_t^{-\varphi} = \lambda_t^{n^M} \psi_t^{hM} s_t + \lambda_t^{n^S} \psi_t^{hS}(1 - s_t) + \lambda_t^c \bar{b}_t, \tag{A.10}$$

$$\lambda_t^{n^M} \psi_t^{hM} = \lambda_t^{n^S} \psi_t^{hS}. \tag{A.11}$$

2 The wage bargaining problem

2.1 Manufacturing sector

The maximization problem is written as,

$$\max_{w_t^M} \left\{ \left(1 - \vartheta^M\right) \ln V_{n^M t}^h + \vartheta^M \ln V_{n^M t}^f \right\}, \tag{A.12}$$

where

$$V_{n^M t}^h = -\Phi l_t^{-\varphi} + \lambda_t^c w_t^M + (1 - \sigma^M) \lambda_t^{n^M}, \tag{A.13}$$

and

$$V_{n^M t}^f = p_t^M (1 - \zeta) \left(\frac{M_t}{n_t^M} \right)^{\frac{1}{\alpha}} - w_t^M + \frac{\left(1 - \sigma^M\right) \kappa^M}{\psi_t^{fM}}. \tag{A.14}$$

The first-order condition is written as,

$$\vartheta^M V_{n^M t}^h = \left(1 - \vartheta^M\right) \lambda_t^c V_{n^M t}^f.$$

Using (A.13) and (A.14), we obtain,

$$\vartheta^M \left(\lambda_t^c w_t^M - \Phi l_t^{-\varphi} + (1 - \sigma^M) \lambda_t^{n^M} \right) = \\ \left(1 - \vartheta^M\right) \lambda_t^c \left(p_t^M (1 - \zeta) \left(\frac{M_t}{n_t^M} \right)^{\frac{1}{\alpha}} - w_t^M + \frac{\left(1 - \sigma^M\right) \kappa^M}{\psi_t^{fM}} \right),$$

$$\vartheta^M \lambda_t^c w_t^M + \vartheta^M \left(-\Phi l_t^{-\varphi} + (1 - \sigma^M) \lambda_t^{n^M} \right) = \\ - (1 - \vartheta^M) \lambda_t^c w_t^M + \left(1 - \vartheta^M\right) \lambda_t^c \left(p_t^M (1 - \zeta) \left(\frac{M_t}{n_t^M} \right)^{\frac{1}{\alpha}} + \frac{\left(1 - \sigma^M\right) \kappa^M}{\psi_t^{fM}} \right),$$

$$\vartheta^M \lambda_t^c w_t^M + (1 - \vartheta^M) \lambda_t^c w_t^M =$$

$$-\vartheta^M\left(-\Phi l_t^{-\varphi}+(1-\sigma^M)\lambda_t^{n^M}\right)+\left(1-\vartheta^M\right)\lambda_t^c\left(p_t^M(1-\zeta)\left(\frac{M_t}{n_t^M}\right)^{\frac{1}{\alpha}}+\frac{\left(1-\sigma^M\right)\kappa^M}{\psi_t^{fM}}\right).$$

The final expression for the wage in the manufacturing sector is,

$$w_t^M=\left(1-\vartheta^M\right)\left(p_t^M(1-\zeta)\left(\frac{M_t}{n_t^M}\right)^{\frac{1}{\alpha}}+\frac{\left(1-\sigma^M\right)\kappa^M}{\psi_t^{fM}}\right)-\frac{\vartheta^M}{\lambda_t^c}\left((1-\sigma^M)\lambda_t^{n^M}-\Phi l_t^{-\varphi}\right).$$

2.2 Service sector

Similarly, the maximization problem is written as,

$$\max_{w_t^S}\left\{\left(1-\vartheta^S\right)\ln V_{n^S t}^h+\vartheta^S\ln V_{n^S t}^f\right\}, \tag{A.15}$$

where

$$V_{n^S t}^h=\lambda_t^c w_t^S-\Phi l_t^{-\varphi}+\left(1-\sigma^S\right)\lambda_t^{n^S}, \tag{A.16}$$

and

$$V_{n^S t}^f=p_t^S(1-\xi)\left(\frac{S_t}{n_t^S}\right)^{\frac{1}{\rho}}-w_t^S+\frac{\left(1-\sigma^S\right)\kappa^S}{\psi_t^{fS}}. \tag{A.17}$$

The first-order condition is written as,

$$\vartheta^S V_{n^S t}^h=\left(1-\vartheta^S\right)\lambda_t^c V_{n^S t}^f.$$

Using (A.16) and (A.17) we obtain,

$$\vartheta^S(\lambda_t^c w_t^S-\Phi l_t^{-\varphi}+\left(1-\sigma^S\right)\lambda_t^{n^S})=\left(1-\vartheta^S\right)\lambda_t^c(p_t^S(1-\xi)\left(\frac{S_t}{n_t^S}\right)^{\frac{1}{\rho}}-w_t^S+\frac{\left(1-\sigma^S\right)\kappa^S}{\psi_t^{fS}}).$$

We perform a simple algebra below,

$$\begin{aligned}&\vartheta^S\lambda_t^c w_t^S+\vartheta^S(-\Phi l_t^{-\varphi}+\left(1-\sigma^S\right)\lambda_t^{n^S})=\\&\qquad-\left(1-\vartheta^S\right)\lambda_t^c w_t^S+\left(1-\vartheta^S\right)\lambda_t^c(p_t^S(1-\xi)\left(\frac{S_t}{n_t^S}\right)^{\frac{1}{\rho}}+\frac{\left(1-\sigma^S\right)\kappa^S}{\psi_t^{fS}}),\end{aligned}$$

or equivalently,

$$\vartheta^S\lambda_t^c w_t^S+\left(1-\vartheta^S\right)\lambda_t^c w_t^S=$$

$$\left(1-\vartheta^S\right)\lambda_t^c(p_t^S(1-\xi)\left(\frac{S_t}{n_t^S}\right)^{\frac{1}{\rho}}+\frac{\left(1-\sigma^S\right)\kappa^S}{\psi_t^{fS}})-\vartheta^S(-\Phi l_t^{-\varphi}+\left(1-\sigma^S\right)\lambda_t^{n^S}),$$

or equivalently,

$$w_t^S\lambda_t^c=\left(1-\vartheta^S\right)\lambda_t^c(p_t^S(1-\xi)\left(\frac{S_t}{n_t^S}\right)^{\frac{1}{\rho}}+\frac{\left(1-\sigma^S\right)\kappa^S}{\psi_t^{fS}})-\vartheta^S(-\Phi l_t^{-\varphi}+\left(1-\sigma^S\right)\lambda_t^{n^S}).$$

The final expression for the equilibrium wage is given by,

$$w_t^S=\left(1-\vartheta^S\right)\left(p_t^S(1-\xi)\left(\frac{S_t}{n_t^S}\right)^{\frac{1}{\rho}}+\frac{\left(1-\sigma^S\right)\kappa^S}{\psi_t^{fS}}\right)-\frac{\vartheta^S}{\lambda_t^c}((1-\sigma^S)\lambda_t^{n^S}-\Phi l_t^{-\varphi}).$$

3 Resource constraint

From the household budget constraint,

$$c_t+i_t=r_tk_t+w_t^Mn_t^M+w_t^Sn_t^S+\bar{b}_tu_t-T_t+\Pi_t^M+\Pi_t^S.$$

We substitute the expressions of the instantaneous profits of firms,

$$\Pi_t^M=p_t^MM_t-w_t^Mn_t^M-r_tk_t^M-\kappa^Mv_t^M,$$

$$\Pi_t^S=p_t^SS_t-w_t^Sn_t^S-r_tk_t^S-\kappa^Sv_t^S,$$

and obtain,

$$c_t+i_t=p_t^MM_t+p_t^SS_t-\kappa^Sv_t^S-\kappa^Mv_t^m.$$

Because of the constant returns to scale and frictionless production of the final good, we have,

$$Y_t=p_t^MM_t+p_t^SS_t.$$

So, we obtain the resource constraint,

$$Y_t=c_t+i_t+\kappa^Sv_t^S+\kappa^Mv_t^m.$$

4 Model Solution

We report below all equations that need to be satisfied in equilibrium.

Labor markets

$$m_t^M = \mu_1(\upsilon_t^M)^{\mu_2}(u_t^M)^{1-\mu_2} \tag{E.1}$$

$$m_t^S = \mu_1(\upsilon_t^S)^{\mu_2}(u_t^S)^{1-\mu_2} \tag{E.2}$$

$$n_{t+1}^M = (1-\sigma^M)n_t^M + m_t^M \tag{E.3}$$

$$n_{t+1}^S = (1-\sigma^S)n_t^S + m_t^S \tag{E.4}$$

$$\psi_t^{hM} \equiv \frac{m_t^M}{u_t^M} \tag{E.5}$$

$$\psi_t^{fM} \equiv \frac{m_t^M}{\upsilon_t^M} \tag{E.6}$$

$$\psi_t^{hS} \equiv \frac{m_t^S}{u_t^S} \tag{E.7}$$

$$\psi_t^{fS} \equiv \frac{m_t^S}{\upsilon_t^S} \tag{E.8}$$

Household

$$c_t^{-\eta} = \beta E_t\left[c_{t+1}^{-\eta}(1-\delta+r_{t+1})\right] \tag{E.9}$$

$$\lambda_t^{n^M} = \beta E_t\left[-\Phi l_{t+1}^{-\varphi} + c_{t+1}^{-\eta}w_{t+1}^M + \lambda_{t+1}^{n^M}(1-\sigma^M)\right] \tag{E.10}$$

$$\lambda_t^{n^S} = \beta E_t\left[-\Phi l_{t+1}^{-\varphi} + c_{t+1}^{-\eta}w_{t+1}^S + \lambda_{t+1}^{n^S}(1-\sigma^S)\right] \tag{E.11}$$

$$\Phi l_t^{-\varphi} = \lambda_t^{n^M}\psi_t^{hM}s_t + \lambda_t^{n^S}\psi_t^{hS}(1-s_t) + \lambda_t^c\bar{b}_t \tag{E.12}$$

$$\lambda_t^{n^M}\psi_t^{hM} = \lambda_t^{n^S}\psi_t^{hS} \tag{E.13}$$

$$n_t^M + n_t^S + u_t + l_t = 1 \tag{E.14}$$

$$u_t^M = s_t u_t \tag{E.15}$$

$$u_t^S = (1 - s_t) u_t \tag{E.16}$$

$$c_t + k_{t+1} = (1 - \delta + r_t) k_t + w_t^M n_t^M + w_t^S n_t^S + \bar{b}_t u_t - T_t + \Pi_t^M + \Pi_t^S \tag{E.17}$$

Capital market clearing

$$k_t = k_t^M + k_t^S \tag{E.18}$$

Firms

$$\Lambda_{t,t+1} = \beta \left(\frac{\lambda_{t+1}^c}{\lambda_t^c} \right) \tag{E.19}$$

$$\frac{\kappa^M}{\psi_t^{fM}} = E_t \Lambda_{t,t+1} \left[p_{t+1}^M (1 - \zeta) \left(\frac{M_{t+1}}{n_{t+1}^M} \right)^{\frac{1}{\alpha}} - w_{t+1}^M + \frac{\left(1 - \sigma^M\right) \kappa^M}{\psi_{t+1}^{fM}} \right] \tag{E.20}$$

$$r_t = p_t^M \cdot \zeta \left(\frac{M_t}{k_t^M} \right)^{\frac{1}{\alpha}} \tag{E.21}$$

$$p_t^M = \gamma \left(\frac{Y_t}{M_t} \right)^{\frac{1}{\chi}} \tag{E.22}$$

$$M_t = \left[\zeta (k_t^M)^{\frac{\alpha - 1}{\alpha}} + (1 - \zeta)(n_t^M)^{\frac{\alpha - 1}{\alpha}} \right]^{\frac{\alpha}{\alpha - 1}} \tag{E.23}$$

$$Y_t = \left[\gamma M_t^{\frac{\chi - 1}{\chi}} + (1 - \gamma) S_t^{\frac{\chi - 1}{\chi}} \right]^{\frac{\chi}{\chi - 1}} \tag{E.24}$$

$$\frac{\kappa^S}{\psi_t^{fS}} = E_t \Lambda_{t,t+1} \left[p_{t+1}^S (1 - \xi) \left(\frac{S_{t+1}}{n_{t+1}^S} \right)^{\frac{1}{\rho}} - w_{t+1}^S + \frac{\left(1 - \sigma^S\right) \kappa^S}{\psi_{t+1}^{fS}} \right] \tag{E.25}$$

$$r_t = p_t^S \cdot \xi \left(\frac{S_t}{k_t^S} \right)^{\frac{1}{\rho}} \tag{E.26}$$

$$p_t^S = (1-\gamma)\left(\frac{Y_t}{S_t}\right)^{\frac{1}{\chi}} \tag{E.27}$$

$$S_t = \left[\xi(k_t^S)^{\frac{\rho-1}{\rho}} + (1-\xi)(n_t^S)^{\frac{\rho-1}{\rho}}\right]^{\frac{\rho}{\rho-1}} \tag{E.28}$$

Wages

$$w_t^S = \left(1-\vartheta^S\right)\left(p_t^S\left(1-\xi\right)\left(\frac{S_t}{n_t^S}\right)^{\frac{1}{\rho}} + \frac{\left(1-\sigma^S\right)\kappa^S}{\psi_t^{fS}}\right) + \frac{\vartheta^S}{\lambda_t^c}(\Phi l_t^{-\varphi} - \left(1-\sigma^S\right)\lambda_t^{n^S}) \tag{E.29}$$

$$w_t^M = \left(1-\vartheta^M\right)\left(p_t^M(1-\zeta)\left(\frac{M_t}{n_t^M}\right)^{\frac{1}{\alpha}} + \frac{\left(1-\sigma^M\right)\kappa^M}{\psi_t^{fM}}\right) + \frac{\vartheta^M}{\lambda_t^c}(\Phi l_t^{-\varphi} - \left(1-\sigma^M\right)\lambda_t^{n^M}) \tag{E.30}$$

5 Steady-state system of equations

$$m^M = \mu_1(\upsilon^M)^{\mu_2}(u^M)^{1-\mu_2} \tag{S.1}$$

$$m^S = \mu_1(\upsilon^S)^{\mu_2}(u^S)^{1-\mu_2} \tag{S.2}$$

$$n^M = \frac{m^M}{\sigma^M} \tag{S.3}$$

$$n^S = \frac{m^S}{\sigma^S} \tag{S.4}$$

$$\psi^{hM} \equiv \frac{m^M}{u^M}, \tag{S.5}$$

$$\psi^{fM} \equiv \frac{m^M}{\upsilon^M}, \tag{S.6}$$

$$\psi^{hS} \equiv \frac{m^S}{u^S}, \tag{S.7}$$

$$\psi^{fS} \equiv \frac{m^S}{\upsilon^S} \tag{S.8}$$

Household

$$r = \frac{1-\beta}{\beta} + \delta \tag{S.9}$$

$$\lambda^{n^M} = \beta \left[-\Phi l^{-\varphi} + c^{-\eta} w^M + \lambda^{n^M} (1 - \sigma^M) \right] \tag{S.10}$$

$$\lambda^{n^S} = \beta \left[-\Phi l^{-\varphi} + c^{-\eta} w^S + \lambda^{n^S} (1 - \sigma^S) \right] \tag{S.11}$$

$$\Phi l^{-\varphi} = \lambda^{n^M} \psi^{hM} s + \lambda^{n^S} \psi^{hS} (1-s) + \lambda^c \bar{b} \tag{S.12}$$

$$\lambda^{n^M} \psi^{hM} = \lambda^{n^S} \psi^{hS} \tag{S.13}$$

$$n^M + n^S + u + l = 1 \tag{S.14}$$

$$u^M = su \tag{S.15}$$

$$u^S = (1-s)u \tag{S.16}$$

$$c = k(r - \delta) + w^M n^M + w^S n^S + \bar{b} u \tag{S.17}$$

Capital market clearing

$$k = k^M + k^S \tag{S.18}$$

Firms

$$\frac{\kappa^S}{\psi^{fS}} = \beta \left[p^S (1 - \xi) \left(\frac{S}{n^S} \right)^{\frac{1}{\rho}} - w^S + \frac{\left(1 - \sigma^S\right) \kappa^S}{\psi^{fS}} \right] \tag{S.19}$$

$$p^S = (1 - \gamma) \left(\frac{Y}{S} \right)^{\frac{1}{\chi}} \tag{S.20}$$

$$S = \left[\xi (k^S)^{\frac{\rho - 1}{\rho}} + (1 - \xi)(n^S)^{\frac{\rho - 1}{\rho}} \right]^{\frac{\rho}{\rho - 1}} \tag{S.21}$$

$$r = p^S \cdot \xi \left(\frac{S}{k^S} \right)^{\frac{1}{\rho}} \tag{S.22}$$

$$\frac{\kappa^M}{\psi_t^{fM}} = \beta\left[p^M(1-\zeta)\left(\frac{M}{n^M}\right)^{\frac{1}{\alpha}} - w^M + \frac{\left(1-\sigma^M\right)\kappa^M}{\psi^{fM}}\right] \tag{S.23}$$

$$r = p^M \cdot \zeta\left(\frac{M}{k^M}\right)^{\frac{1}{\alpha}} \tag{S.24}$$

$$p^M = \gamma\left(\frac{Y}{M}\right)^{\frac{1}{\chi}} \tag{S.25}$$

$$M = \left[\zeta(k^M)^{\frac{\alpha-1}{\alpha}} + (1-\zeta)(n^M)^{\frac{\alpha-1}{\alpha}}\right]^{\frac{\alpha}{\alpha-1}} \tag{S.26}$$

Wages

$$w^S = \left(1-\vartheta^S\right)\left(p^S\left(1-\xi\right)\left(\frac{S}{n^S}\right)^{\frac{1}{\rho}} + + \frac{\left(1-\sigma^S\right)\kappa^S}{\psi^{fS}}\right) + \frac{\vartheta^S}{\lambda^c}(\Phi l^{-\varphi} - \left(1-\sigma^S\right)\lambda^{n^S}) \tag{S.27}$$

$$w^M = \left(1-\vartheta^M\right)\left(p^M(1-\zeta)\left(\frac{M}{n^M}\right)^{\frac{1}{\alpha}} + \frac{\left(1-\sigma^M\right)\kappa^M}{\psi^{fM}}\right) + \frac{\vartheta^M}{\lambda^c}(\Phi l^{-\varphi} - \left(1-\sigma^M\right)\lambda^{n^M}) \tag{S.28}$$

5.1 Steady state - simplified system

Given the unknowns u, v^M, v^S, k^S, k^M, s, the following variables are determined sequentially:

$$r = \frac{1-\beta}{\beta} + \delta \tag{SS.1}$$

$$u^M = su \tag{SS.2}$$

$$u^S = (1-s)u \tag{SS.3}$$

$$m^M = \mu_1(v^M)^{\mu_2}(u^M)^{1-\mu_2} \tag{SS.4}$$

$$m^S = \mu_1(v^S)^{\mu_2}(u^S)^{1-\mu_2} \tag{SS.5}$$

$$n^M = \frac{m^M}{\sigma^M} \tag{SS.6}$$

$$n^S = \frac{m^S}{\sigma^S} \tag{SS.7}$$

$$\psi^{hM} \equiv \frac{m^M}{u^M}, \tag{SS.8}$$

$$\psi^{fM} \equiv \frac{m^M}{\upsilon^M}, \tag{SS.9}$$

$$\psi^{hS} \equiv \frac{m^S}{u^S}, \tag{SS.10}$$

$$\psi^{fS} \equiv \frac{m^S}{\upsilon^S} \tag{SS.11}$$

$$l = 1 - u - n^M - n^S \tag{SS.12}$$

$$S = \left[\xi (k^S)^{\frac{\rho-1}{\rho}} + (1-\xi)(n^S)^{\frac{\rho-1}{\rho}}\right]^{\frac{\rho}{\rho-1}} \tag{SS.13}$$

$$M = \left[\zeta (k^M)^{\frac{\alpha-1}{\alpha}} + (1-\zeta)(n^M)^{\frac{\alpha-1}{\alpha}}\right]^{\frac{\alpha}{\alpha-1}} \tag{SS.14}$$

$$Y = \left[\gamma M^{\frac{\chi-1}{\chi}} + (1-\gamma) S^{\frac{\chi-1}{\chi}}\right]^{\frac{\chi}{\chi-1}} \tag{SS.15}$$

$$p^S = (1-\gamma)\left(\frac{Y}{S}\right)^{\frac{1}{\chi}} \tag{SS.16}$$

$$p^M = \gamma\left(\frac{Y}{M}\right)^{\frac{1}{\chi}} \tag{SS.17}$$

The wages of two sectors are obtained from the inverted FOCs with respect to sectoral employment:

$$w^S = p^S (1-\xi)\left(\frac{S}{n^S}\right)^{\frac{1}{\rho}} - \frac{\kappa^S}{\psi^{fS}\beta} + \frac{(1-\sigma^S)\kappa^S}{\psi^{fS}} \tag{SS.18}$$

$$w^M = p^M (1-\zeta)\left(\frac{M}{n^M}\right)^{\frac{1}{\alpha}} - \frac{\kappa^M}{\psi^{fM}\beta} + \frac{\left(1-\sigma^M\right)\kappa^M}{\psi^{fM}} \tag{SS.19}$$

$$\bar{b} = \varpi \frac{(w^m n^M + w^S n^S)}{n^M + n^S} \tag{SS.20}$$

$$T = \bar{b}u \tag{SS.21}$$

$$k = k^M + k^S \tag{SS.22}$$

$$c = k(r - \delta) + w^M n^M + w^S n^S + \bar{b}u - T + \Pi^M + \Pi^S \tag{SS.23}$$

$$\lambda^c = c^{-\eta} \tag{SS.24}$$

$$\lambda^{n^M} = \frac{\beta}{1 - \beta(1 - \sigma)} \left(c^{-\eta} w^M - \Phi l^{-\varphi}\right) \tag{SS.25}$$

$$\lambda^{n^S} = \frac{\beta}{1 - \beta(1 - \sigma)} \left(c^{-\eta} w^S - \Phi l^{-\varphi}\right) \tag{SS.26}$$

To solve for the unknowns, we numerically find the solution to the following system of equations, in which we use expressions (S.1) to (S.25).

$$\Phi l^{-\varphi} = \lambda^{n^M} \psi^{hM} s + \lambda^{n^S} \psi^{hS}(1 - s) + \lambda^c \bar{b} \tag{SS.27}$$

$$p^M \cdot \zeta \left(\frac{M}{k^M}\right)^{\frac{1}{\alpha}} - r = 0 \tag{SS.28}$$

$$p^S \cdot \xi \left(\frac{S}{k^S}\right)^{\frac{1}{\rho}} - r = 0 \tag{SS.29}$$

$$\frac{\kappa^S}{\psi^{fS}} = \beta \left[p^S(1 - \xi) \left(\frac{S}{n^S}\right)^{\frac{1}{\rho}} - w^S + \frac{\left(1 - \sigma^S\right) \kappa^S}{\psi^{fS}}\right] \tag{SS.30}$$

$$\frac{\kappa^M}{\psi_t^{fM}} = \beta \left[p^M(1 - \zeta) \left(\frac{M}{n^M}\right)^{\frac{1}{\alpha}} - w^M + \frac{\left(1 - \sigma^M\right) \kappa^M}{\psi^{fM}}\right] \tag{SS.31}$$

$$w^S = \left(1 - \vartheta^S\right) \left(p^S(1 - \xi) \left(\frac{S}{n^S}\right)^{\frac{1}{\rho}} + \frac{\left(1 - \sigma^S\right) \kappa^S}{\psi^{fS}}\right) + \frac{\vartheta^S}{\lambda^c} (\Phi l^{-\varphi} - \left(1 - \sigma^S\right) \lambda^{n^S}) \tag{SS.32}$$

$$w^M = \left(1-\vartheta^M\right)\left(p^M(1-\zeta)\left(\frac{M}{n^M}\right)^{\frac{1}{\alpha}} + \frac{\left(1-\sigma^M\right)\kappa^M}{\psi^{fM}}\right) + \frac{\vartheta^M}{\lambda^c}(\Phi l^{-\varphi} - \left(1-\sigma^M\right)\lambda^{n^M}) \tag{SS.33}$$

$$\lambda^{n^M}\psi^{hM} = \lambda^{n^S}\psi^{hS} \tag{SS.34}$$

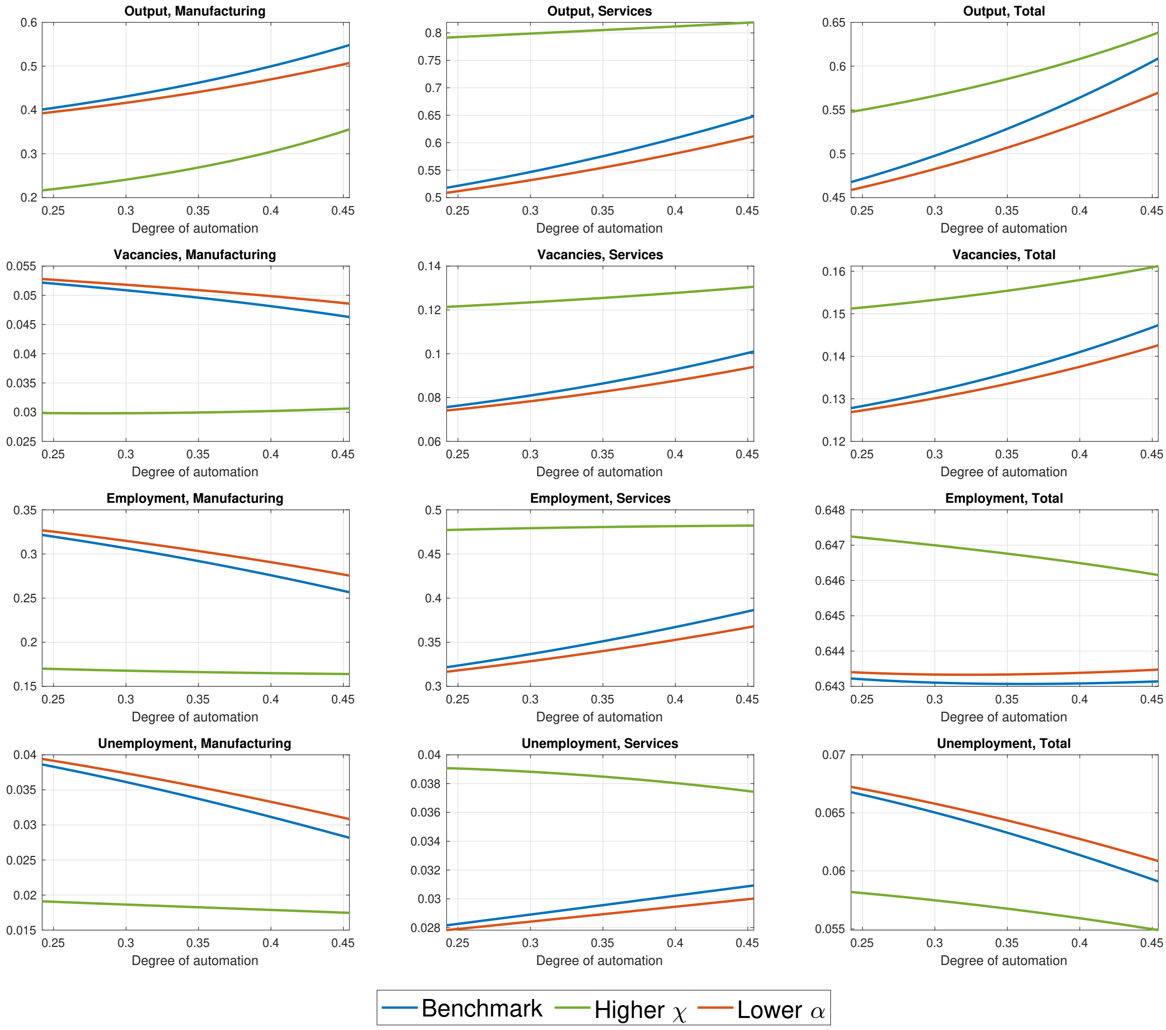

Figure A.1: Steady-state effects of automation in a two-sector economy: Different elasticities of substitution between capital and labor ($\alpha = 0.7$) and between the two goods ($\chi = 1.5$)

Note: The y-axis shows steady-state levels.

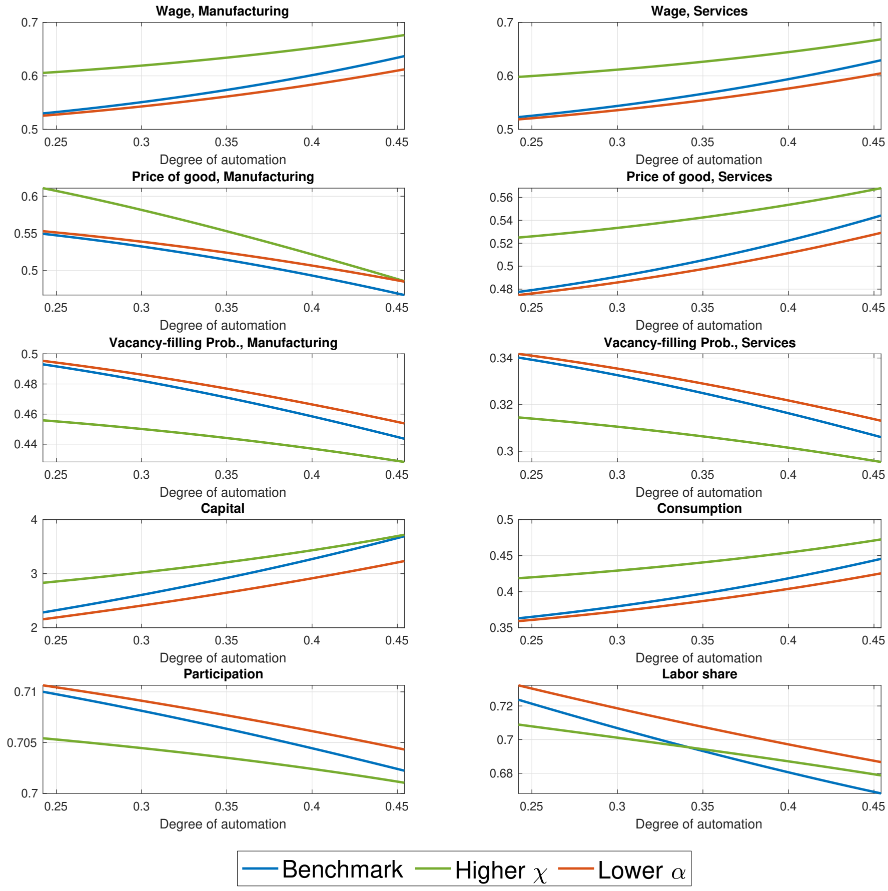

Figure A.2: Steady-state effects of automation in a two-sector economy: Different elasticities of substitution between capital and labor ($\alpha = 0.7$) and between the two goods ($\chi = 1.5$) (continued)

Note: The y-axis shows steady-state levels.

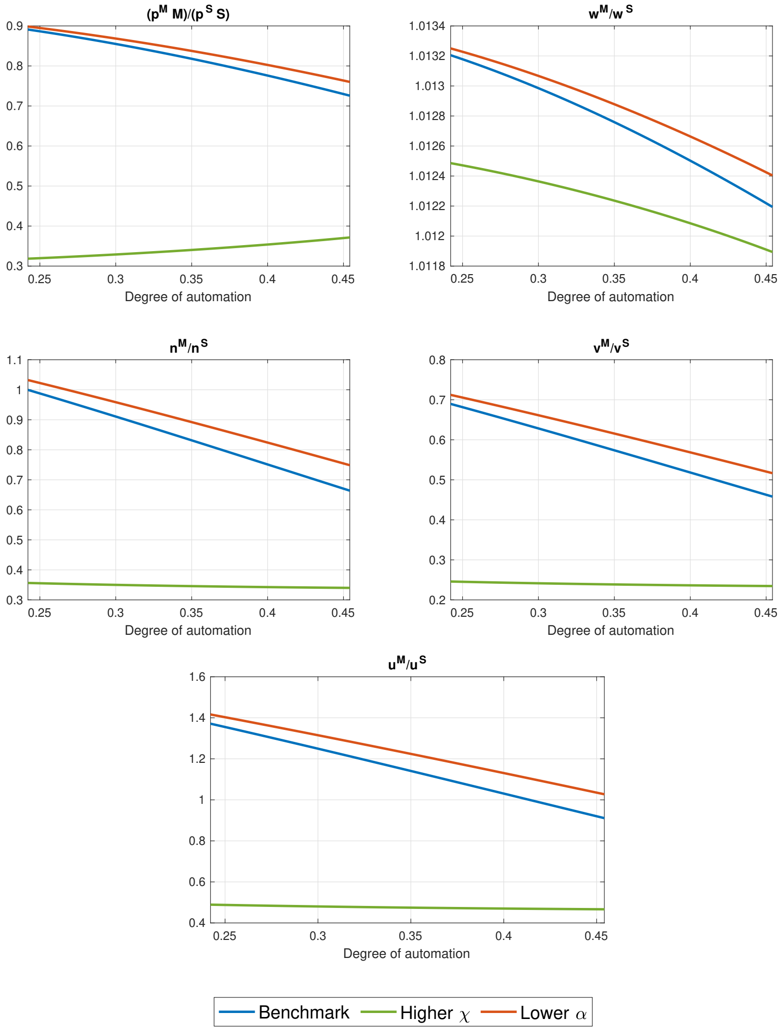

Figure A.3: Steady-state effects of automation on key ratios in a two-sector economy: Different elasticities of substitution between capital and labor ($\alpha = 0.7$) and between the two goods ($\chi = 1.5$)

Note: The y-axis shows steady-state levels.

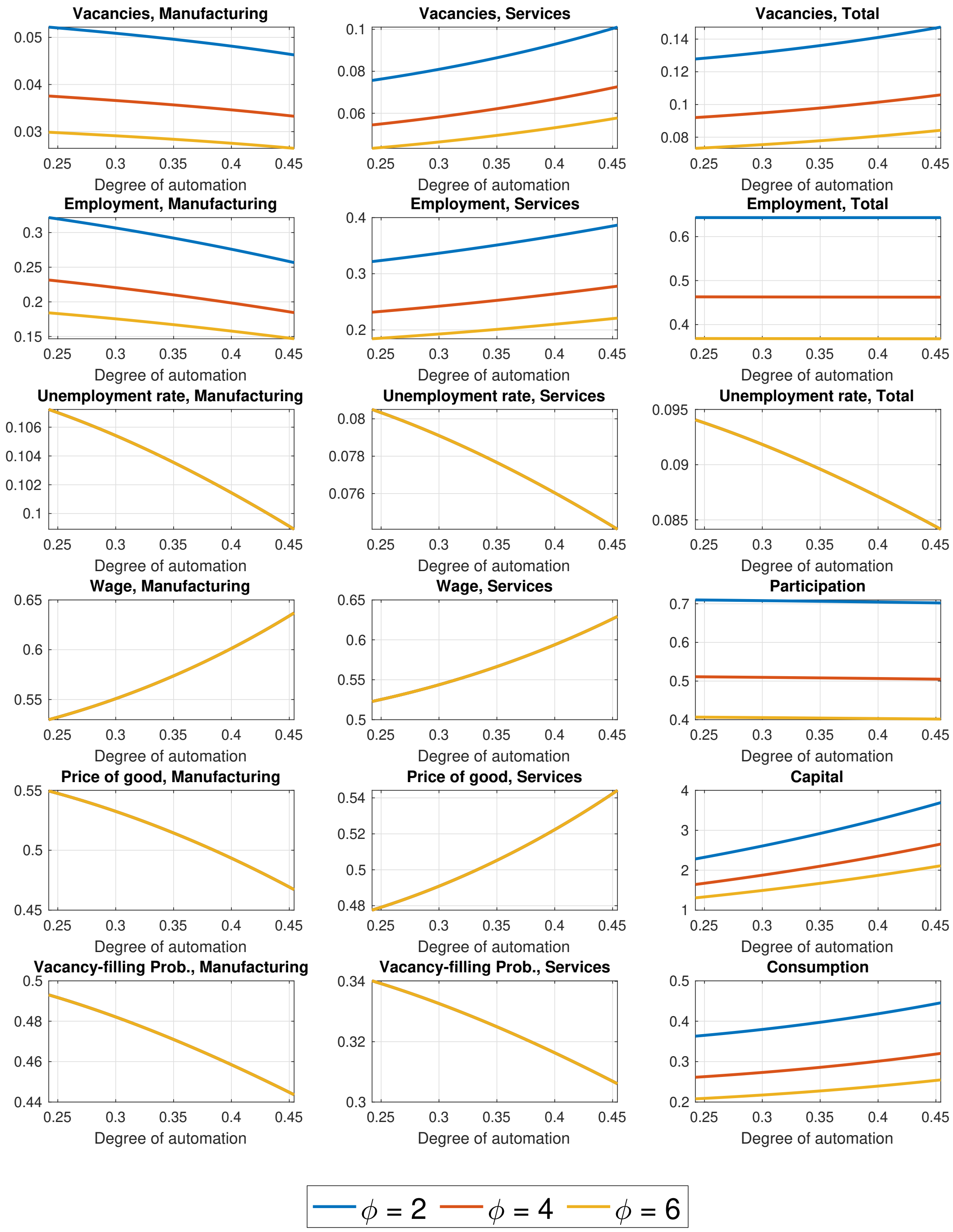

Figure A.4: Steady-state effects of automation in a two-sector economy: Different values of the Frisch elasticity

Note: The y-axis shows steady-state levels.

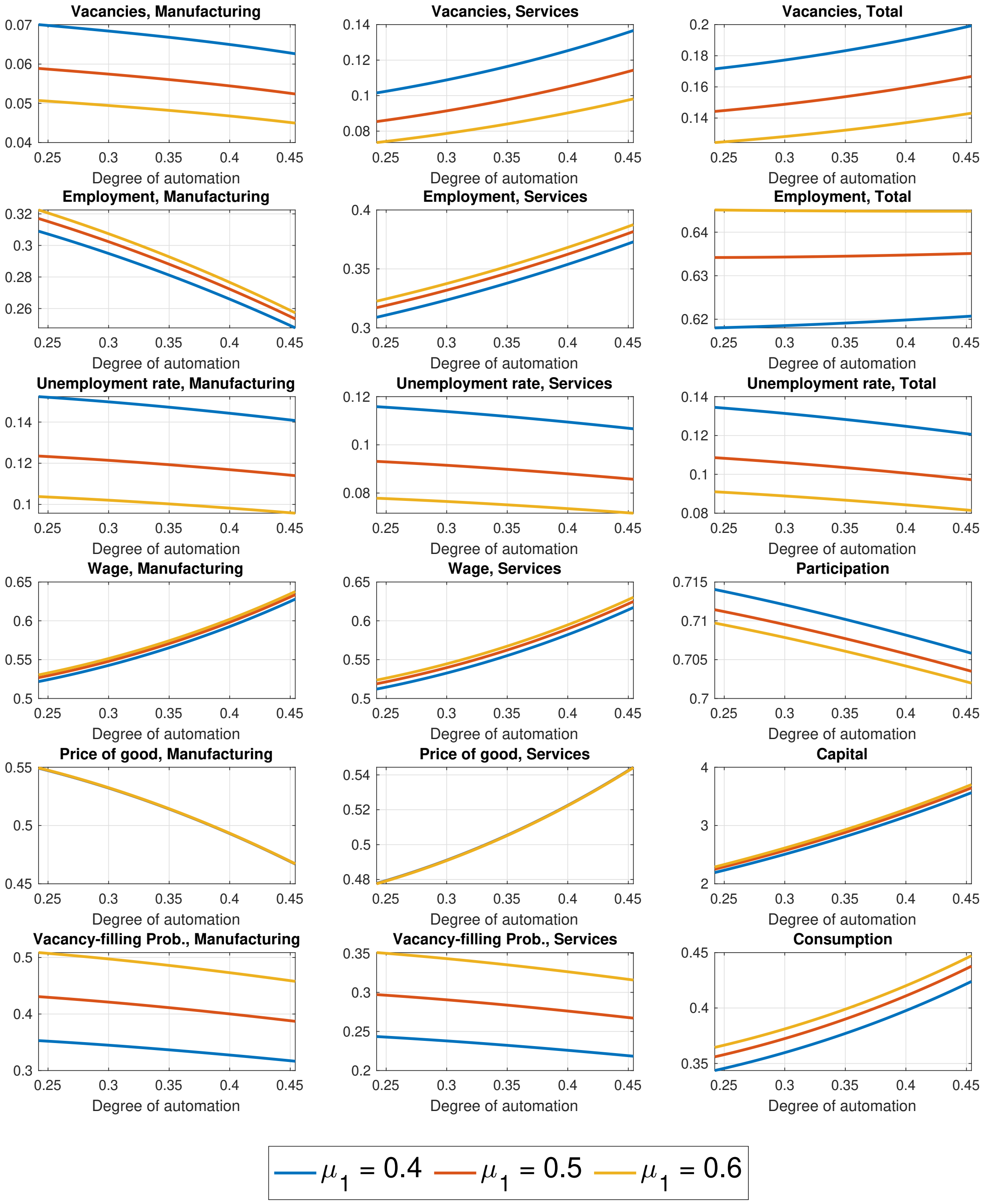

Figure A.5: Steady-state effects of automation in a two-sector economy: Different values of matching efficiency

Note: The y-axis shows steady-state levels.

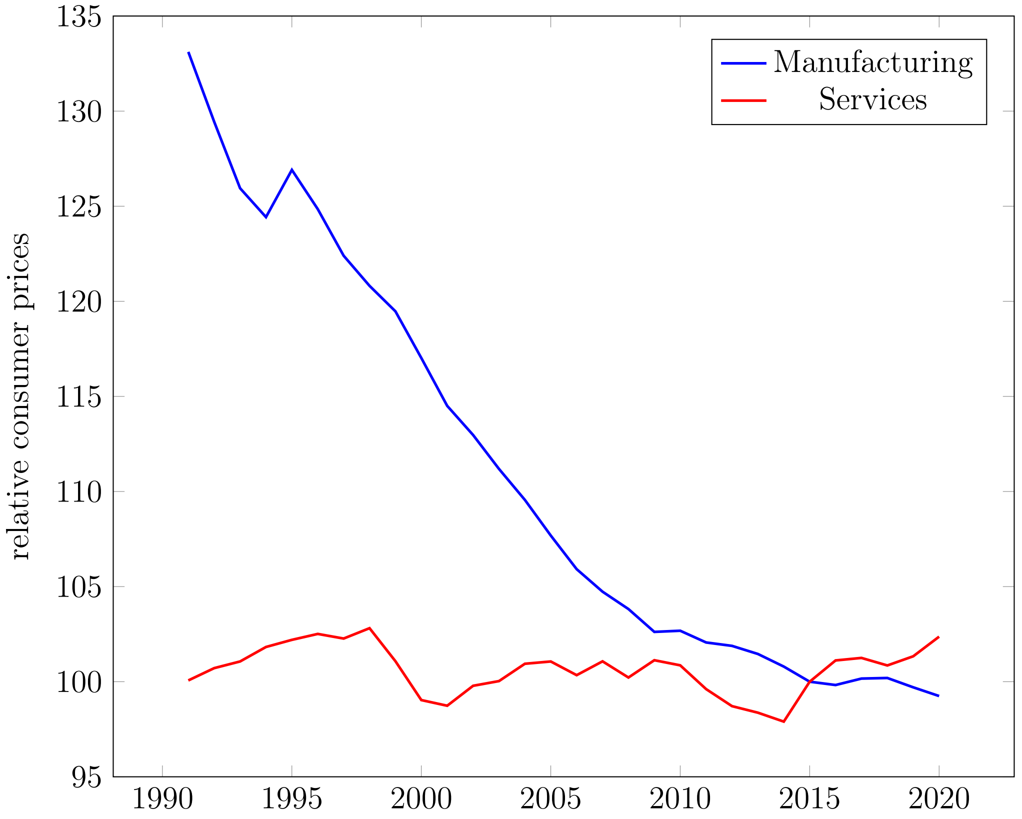

Figure A.6: Price Indexes for Manufacturing and Service goods normalized by the aggregate CPI (2015=100)

Note: We calculate the relative prices using the consumption basket weights and price indexes for goods and services from the Federal Statistical Office (Destatis) to match the definitions of the two sectors used throughout the paper. We normalize the indexes by the aggregate CPI (2015=100) representing the price of the numéraire good.
